# Systemic insecticides (neonicotinoids and fipronil): trends, uses, mode of action and metabolites

**DOI:** 10.1007/s11356-014-3470-y

**Published:** 2014-09-19

**Authors:** N. Simon-Delso, V. Amaral-Rogers, L. P. Belzunces, J. M. Bonmatin, M. Chagnon, C. Downs, L. Furlan, D. W. Gibbons, C. Giorio, V. Girolami, D. Goulson, D. P. Kreutzweiser, C. H. Krupke, M. Liess, E. Long, M. McField, P. Mineau, E. A. D. Mitchell, C. A. Morrissey, D. A. Noome, L. Pisa, J. Settele, J. D. Stark, A. Tapparo, H. Van Dyck, J. Van Praagh, J. P. Van der Sluijs, P. R. Whitehorn, M. Wiemers

**Affiliations:** 1 Environmental Sciences, Copernicus Institute, Utrecht University, Heidelberglaan 2, 3584 CS Utrecht, The Netherlands; 2 Beekeeping Research and Information Centre (CARI), Place Croix du Sud 4, 1348 Louvain-la-Neuve, Belgium; 3 Buglife, Bug House, Ham Lane, Orton Waterville, PE2 5UU Peterborough, UK; 4 INRA, UR 406 Abeilles & Environnement, Laboratoire de Toxicologie Environnementale, Site Agroparc, 84000 Avignon, France; 5 Centre National de la Recherche Scientifique, Centre de Biophysique Moléculaire, rue Charles Sadron, 45071 Orléans Cedex 02, France; 6 Université du Québec À Montréal, Département des sciences biologiques, Case Postale 8888, succursale Centre-ville, Montréal, Québec Canada H3C 3P8; 7 Haereticus Environmental Laboratory, P.O. Box 92, Clifford, VA 24533 USA; 8 Veneto Agricoltura, Legnaro, PD Italy; 9 Centre for Conservation Science (RSPB), The Lodge, Sandy, Bedfordshire SG19 2DL UK; 10 Department of Chemistry, University of Cambridge, Lensfield Road, CB2 1EW Cambridge, UK; 11 Dipartimento di Agronomia Animali Alimenti Risorse Naturali e Ambiente, Università degli Studi di Padova, Agripolis, viale dell’Università 16, 35020 Legnaro, Padova Italy; 12 School of Life Sciences, University of Sussex, Brighton, BN1 9RH UK; 13 Canadian Forest Service, Natural Resources Canada, 1219 Queen Street East, Sault Ste Marie, ON Canada P6A 2E5; 14 Department of Entomology, Purdue University, West Lafayette, IN USA; 15 Department of System Ecotoxicology, Helmholtz Centre for Environmental Research - UFZ, 04318 Leipzig, Germany; 16 Healthy Reefs for Healthy People Initiative, Smithsonian Institution, Belize City, Belize; 17 Pierre Mineau Consulting, 124 Creekside Drive, Salt Spring Island, V8K 2E4 Canada; 18 Laboratory of Soil Biology, University of Neuchatel, Rue Emile Argand 11, 2000 Neuchatel, Switzerland; 19 Jardin Botanique de Neuchâtel, Chemin du Perthuis-du-Sault 58, 2000 Neuchâtel, Switzerland; 20 Department of Biology and School of Environment and Sustainability, University of Saskatchewan, 112 Science Place, Saskatoon, SK S7N 5E2 Canada; 21 Kijani, Oud Blaricumerweg 36b, 1411JT Naarden, The Netherlands; 22 UFZ, Helmholtz Centre for Environmental Research, Department of Community Ecology, Theodor-Lieser-Str. 4, 06120 Halle, Germany; 23 German Centre for Integrative Biodiversity Research (iDiv), Halle-Jena-Leipzig, Deutscher Platz 5e, 04103 Leipzig, Germany; 24 Puyallup Research and Extension Centre, Washington State University, Puyallup, WA 98371 USA; 25 Dipartimento di Scienze Chimiche, Università degli Studi di Padova, via Marzolo 1, 35131 Padova, Italy; 26 Behavioural Ecology and Conservation Group, Biodiversity Research Centre, Université Catholique de Louvain (UCL), Croix du Sud 4-5 bte L7.07.04, 1348 Louvain-la-Neuve, Belgium; 27 Scientific Advisor, Hassellstr. 23, 29223 Celle, Germany; 28 Centre for the Study of the Sciences and the Humanities, University of Bergen, Postboks 7805, 5020 Bergen, Norway; 29 School of Natural Sciences, University of Stirling, Stirling, FK9 4LA UK

**Keywords:** Neonicotinoid, Fipronil, Trends, Mechanism of action, Agriculture, Seed treatment, Systemic insecticides, Metabolites

## Abstract

Since their discovery in the late 1980s, neonicotinoid pesticides have become the most widely used class of insecticides worldwide, with large-scale applications ranging from plant protection (crops, vegetables, fruits), veterinary products, and biocides to invertebrate pest control in fish farming. In this review, we address the phenyl-pyrazole fipronil together with neonicotinoids because of similarities in their toxicity, physicochemical profiles, and presence in the environment. Neonicotinoids and fipronil currently account for approximately one third of the world insecticide market; the annual world production of the archetype neonicotinoid, imidacloprid, was estimated to be ca. 20,000 tonnes active substance in 2010. There were several reasons for the initial success of neonicotinoids and fipronil: (1) there was no known pesticide resistance in target pests, mainly because of their recent development, (2) their physicochemical properties included many advantages over previous generations of insecticides (i.e., organophosphates, carbamates, pyrethroids, etc.), and (3) they shared an assumed reduced operator and consumer risk. Due to their systemic nature, they are taken up by the roots or leaves and translocated to all parts of the plant, which, in turn, makes them effectively toxic to herbivorous insects. The toxicity persists for a variable period of time—depending on the plant, its growth stage, and the amount of pesticide applied. A wide variety of applications are available, including the most common prophylactic non-Good Agricultural Practices (GAP) application by seed coating. As a result of their extensive use and physicochemical properties, these substances can be found in all environmental compartments including soil, water, and air. Neonicotinoids and fipronil operate by disrupting neural transmission in the central nervous system of invertebrates. Neonicotinoids mimic the action of neurotransmitters, while fipronil inhibits neuronal receptors. In doing so, they continuously stimulate neurons leading ultimately to death of target invertebrates. Like virtually all insecticides, they can also have lethal and sublethal impacts on non-target organisms, including insect predators and vertebrates. Furthermore, a range of synergistic effects with other stressors have been documented. Here, we review extensively their metabolic pathways, showing how they form both compound-specific and common metabolites which can themselves be toxic. These may result in prolonged toxicity. Considering their wide commercial expansion, mode of action, the systemic properties in plants, persistence and environmental fate, coupled with limited information about the toxicity profiles of these compounds and their metabolites, neonicotinoids and fipronil may entail significant risks to the environment. A global evaluation of the potential collateral effects of their use is therefore timely. The present paper and subsequent chapters in this review of the global literature explore these risks and show a growing body of evidence that persistent, low concentrations of these insecticides pose serious risks of undesirable environmental impacts.

## Introduction

Neonicotinoids and the phenyl-pyrazole fipronil are insecticides with systemic properties. Their physicochemical characteristics, mainly assessed in terms of their octanol water partition coefficient (*K*_ow_) and dissociation constant (pKa), enable their entrance into plant tissues and their translocation to all its parts (Bromilow and Chamberlain 1995; Bonmatin et al. 2014). Regardless of the manner of application and route of entry to the plant, they translocate throughout all plant tissues making them toxic to any insects (and potentially other organisms) that feed upon the plant. This protects the plant from direct damage by herbivorous (mainly sap feeding) insects and indirectly from damage by plant viruses that are transmitted by insects. The discovery of imidacloprid by Shinzo Kagabu, and its subsequent market introduction in 1991, started the era of the neonicotinoid class of insecticides (Tomizawa and Casida 2011). Imidacloprid was followed in 1999 by thiamethoxam (Maienfisch et al. 2001a) and clothianidin, which is a metabolite of thiamethoxam (Meredith et al. 2002). Over the following two decades, neonicotinoids have become the most widely used insecticides of the five major chemical classes (the others being organophosphates, carbamates, phenyl-pyrazoles, and pyrethroids) on the global market (Jeschke and Nauen 2008; Jeschke et al. 2011; Casida and Durkin 2013).

The French company Rhône-Poulenc Agro (now Bayer CropScience) discovered and developed fipronil between 1985 and 1987 (Tingle et al. 2003), reaching the market in 1993 (Tomlin 2000). It is noteworthy that substances belonging to the phenyl-pyrazole family have in principal herbicidal effects, whereas fipronil is a potent insecticide.

By the 1980s, many pest insects had developed resistance to the organophosphates, carbamates, and pyrethroids then on the market (Georghiou and Mellon 1983; Denholm et al. 1998; Alyokhin et al. 2008). Set against this background of increased resistance to existing insecticides, the neonicotinoid and fipronil were presented as having several key attributes that led to their rapid adoption in both agricultural and urban environments. These included the following: lower binding efficiencies to vertebrate compared to invertebrate receptors, indicating selective toxicity to arthropods, high persistence, systemic nature, versatility in application (especially as seed treatments), high water solubility, and assumed lower impacts on fish and other vertebrates.

The binding sites of neonicotinoids to nicotinic acetylcholine receptors (nAChRs) and fipronil to γ-aminobutiric acid (GABA) receptors in the nervous systems of vertebrates are different from those in insects. In general, vertebrates have lower numbers of nicotinic receptors with high affinity to neonicotinoids, which is why neonicotinoids generally show a priori higher toxicity to invertebrates than vertebrates (including human, e.g., USEPA 2003a; Tomizawa and Casida 2003; Tomizawa and Casida 2005; Liu et al. 2010; Van der Sluijs et al. 2013). Similarly, the binding of fipronil to insect GABA receptors is tighter than that observed for vertebrate receptors (Cole et al. 1993; Grant et al. 1998; Hainzl et al. 1998; Ratra and Casida 2001; Ratra et al. 2001; Narahashi et al. 2010). This, combined with the frequent use on neonicotinoids and fipronil in seed/soil treatments rather than sprays, is supposed to make them comparatively safe for agricultural workers. This is in contrast to some of the alternatives that they have replaced, such as organophosphates and carbamates (Marrs 1993). Neonicotinoids and fipronil are also relatively persistent, offering the potential for long-term crop protection activity. The half-lives of these compounds in aerobic soil conditions can vary widely, but are measured in months or longer (e.g., 148–6,931 days for clothianidin; USEPA 2003a; Gunasekara et al. 2007; Goulson 2013; Sánchez-Bayo and Hyne 2014). Extensive information about the physicochemical characteristics of neonicotinoids and fipronil can be found in Bonmatin et al. (2014), together with information about their environmental fate.

Arguably, however, it is the systemic nature of these insecticides that has made them so successful. Irrespective of their mode of application, neonicotinoids become distributed throughout the plant, including the apices of new vegetation growth, making them particularly effective against sucking pests, both above ground and below. Although it is not a neonicotinoid, fipronil also acts systemically mainly when it is co-formulated with polymers to increase its systemic activity (Dieckmann et al. 2010a; Dieckmann et al. 2010b; Dieckmann et al. 2010c). Neonicotinoids and fipronil belong to a wide family of substances jointly referred to as the “systemic insecticides” due to their systemic properties, some carbamate and organophosphorus substances, however, can also act systemically (Sanchez-Bayo et al. 2013). Neonicotinoid and fipronil should theoretically not target organisms lacking nervous systems, such as protists, prokaryotes, and plants. Very little research has been done on these non-target organisms and the ecosystem functions they are responsible for. Nevertheless, some studies have revealed negative effects: for example, a negative effect of fipronil on soil microorganisms was suggested as a possible cause for the slower (ca. four-fold) degradation of this pesticide at high vs. low application in Australian soils (Ying and Kookana 2006).

**Fig. 1:**
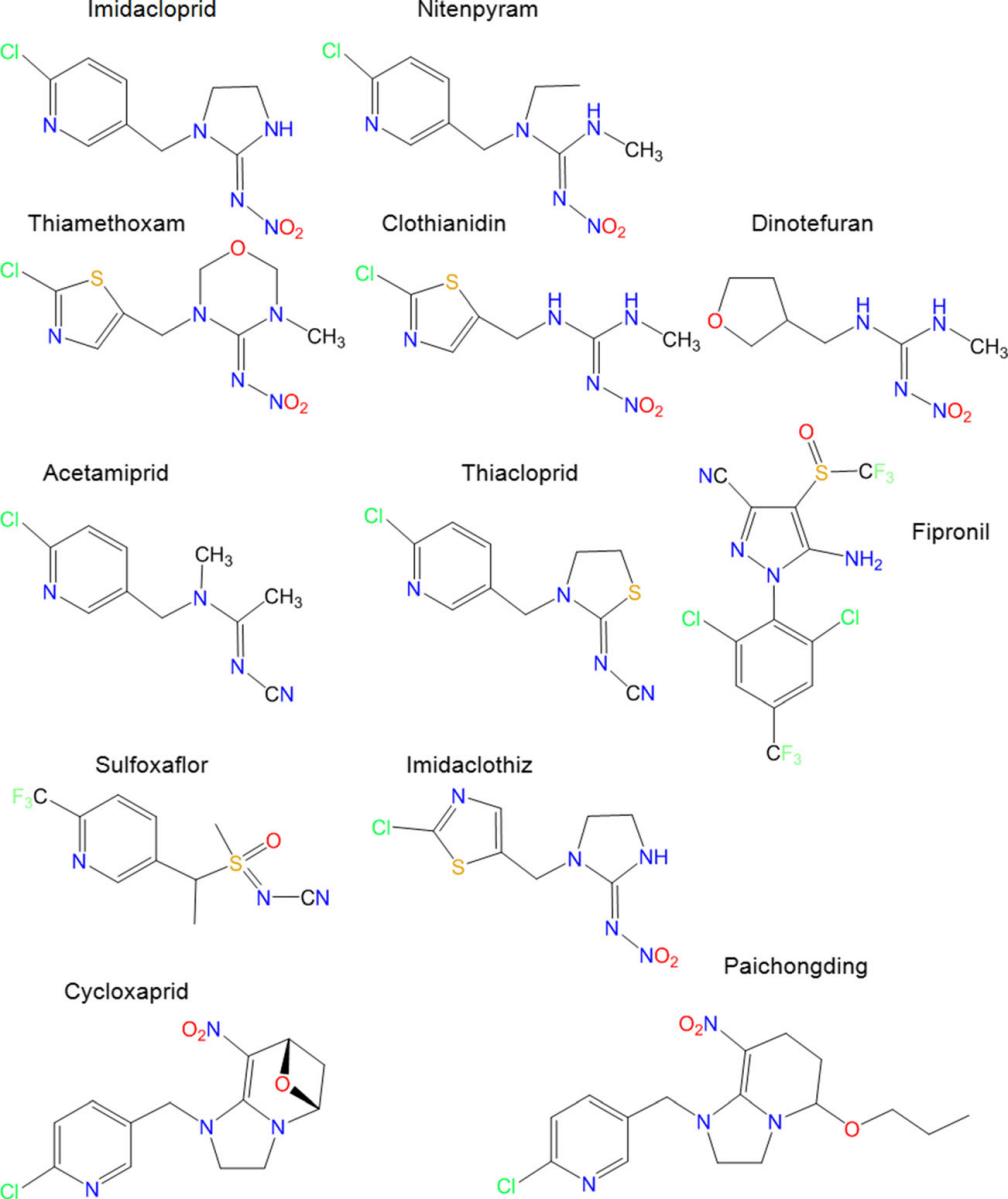
Common names and molecular structures of the systemic insecticides

Seven separate neonicotinoid compounds are available commercially worldwide (Jeschke et al. 2011). These are imidacloprid and thiacloprid (developed by Bayer CropScience), clothianidin (Bayer CropScience and Sumitomo), thiamethoxam (Syngenta), acetamiprid (Nippon Soda), nitenpyram (Sumitomo), and dinotefuran (Mitsui Chemicals). An eighth compound, sulfoxaflor (Zhu et al. 2010), has recently come onto the market in China (Shao et al. 2013b) and the USA (Dow Agro Sciences 2013; USEPA 2013) and has been reviewed by the European Food Safety Authority (EFSA) for approval in the European Union (EFSA 2014). In China, new neonicotinoid compounds are being developed and tested (e.g., guadipyr and huanyanglin), and are nearing market release (Shao et al. 2013b; Shao et al. 2013b). Some of these novel neonicotinoids are the *cis*-neonicotinoids, which are isomers of neonicotinoids in which the nitro or cyano group are in the *cis*, rather than *trans*, orientation. It is well known that *trans* and *cis* isomers can differ markedly in their toxicity. More than 600 *cis*-neonicotinoid compounds have already been synthesized, two of which, paichongding and cycloxaprid (Shao et al. 2013a), might also soon be available on the Chinese market; both are highly effective against Homoptera and Lepidoptera. Through hydrolysis, cycloxaprid forms imidacloprid within the plant, thereby acting as a time-released imidacloprid source, prolonging the protection of the crop. The molecular structures of these systemic pesticides are reported in Fig. 1.

Neonicotinoids are active against a broad spectrum of economically important crop pests, including Aphidae (aphids), Aleyrodidae (whitefly), Cicadellidae (leafhoppers), Chrysomelidae (among others western corn rootworm), Elateridae (wireworms), Fulgoroidea (planthoppers), Pseudococcidae (mealybugs), and phytophagous mites (Elbert et al. 2008; Jeschke et al. 2011). Some of these groups (e.g., aphids) can also transmit viruses, so neonicotinoids can also contribute to the control of insect vectors of crop viral diseases. However, their broad spectrum leads to undesirable effects on non-target insects (Balança and de Visscher 1997; Sánchez-Bayo and Goka 2006; Maini et al. 2010; Lanzoni et al. 2012; Hayasaka et al. 2012a, b; Lu et al. 2012; Fogel et al. 2013; Goulson 2013; Matsumoto 2013; Sanchez-Bayo et al. 2013; Van der Sluijs et al. 2013; Lu et al. 2014; Feltham et al. 2014; Bonmatin et al. 2014; Pisa et al. 2014). Pisa et al. (2014) focus specifically on the undesirable effects of neonicotinoids and fipronil on non-target invertebrates.

## Global growth in the insecticide market

In 1990, the global insecticide market was dominated by carbamates, organophosphates, and pyrethroids. By 2008, one quarter of the insecticide market was neonicotinoid (rising to 27 % in 2010; Casida and Durkin 2013), and nearly 30 % was neonicotinoid and fipronil combined, with the other classes correspondingly reduced (Jeschke et al. 2011). In the same year, imidacloprid became the world’s largest selling insecticide, and second largest selling pesticide (glyphosate was the largest; Pollack 2011) with registered uses for over 140 crops in 120 countries (Jeschke et al. 2011). Neonicotinoids are now in widespread use for a wide variety of crops worldwide.

By 2009, the global neonicotinoid market was worth US $2.63 billion (Jeschke et al. 2011). Imidacloprid accounted for the greatest proportion (41.5 %) of this, and was worth US $1.09 billion, with—in decreasing order of market share—thiamethoxam, clothianidin, acetamiprid, thiacloprid, dinotefuran, and nitenpyram worth US $0.63, 0.44, 0.28, 0.11, 0.08, and 0.008 billion, respectively. Over the period 2003–2009, sales of individual neonicotinoid products (with the single exception of nitenpyram) rose by between 1.6- and 14.6-fold, with total sales across all products rising 2.45-fold (Table 1).

**Table 1:** Growth in global annual turnover (US $ million) of neonicotinoid insecticides. Sales figures for 2003, 2005 & 2007 taken from http://www.agropages.com/BuyersGuide/category/ Neonicotinoid-Insecticide-Insight.html. Sales figures for 2009, and number of crop uses taken from (Jeschke et al. 2011). Products sorted by rank of sales in 2009

Product	Crop uses	Company	2003	2005	2007	2009
imidacloprid	140	Bayer CropScience	665	830	840	1091
thiamethoxam	115	Syngenta	215	359	455	627
clothianidin	40	Sumitomo//Bayer CS	<30	162	365	439
acetamiprid	60	Nippon Soda	60	95	130	276
thiacloprid	50	Bayer CropScience	<30	55	80	112
dinotefuran	35	Mitsui Chemicals	<30	40	60	79
nitenpyram	12	Sumitomo	45	<10	<10	8

According to one estimate, ca. 5,450 tonnes of imidacloprid were sold worldwide in 2008 (Pollack 2011). A separate study estimated that ca. 20,000 tonnes of imidacloprid were produced globally in 2010 (CCM International 2011). This difference may reflect real growth, but may also be because imidacloprid became generic (off-patent) in 2006 (Jeschke et al. 2011), and/or because the estimates differ in the way they were measured, and what they include (e.g., agrochemicals and/or veterinary products, etc.; whether seed treatment is considered as insecticidal or not). Of the estimated 20,000 tonnes, 13,620 tonnes were produced in China (CCM International 2011). Shao et al. (2013b) similarly estimate that China currently produces 14,000 tonnes of imidacloprid annually, exporting 8,000 tonnes. Considering these figures, the estimation of CCM International 2011 seems realistic.

More recently, imidacloprid has been replaced by thiamethoxam and clothianidin in some parts of the world. Consequently, the worldwide sales of thiamethoxam reached US $1 billion in 2011 (Syngenta 2012), and US $1.1 billion in 2012 (Syngenta 2013). In the USA, clothianidin is now registered for use on 146 agricultural crops, and between 2009 and 2011 was applied to about 46 million acres (18.6 million ha) of these crops annually, of which 45 million (18.2 million ha) was corn (maize), *Zea mays* (Brassard 2012). In the USA, the use of clothianidin in 2011 is estimated to be 818 tonnes with corn accounting for 95 % of that use; imidacloprid 811 tonnes (2011) with soybeans and cotton accounting for 60 % of that use; and thiamethoxam 578 tonnes (2011) with soybeans, corn, and cotton accounting for 85 % of that use (US Geological Survey 2014).

**Fig. 2:**
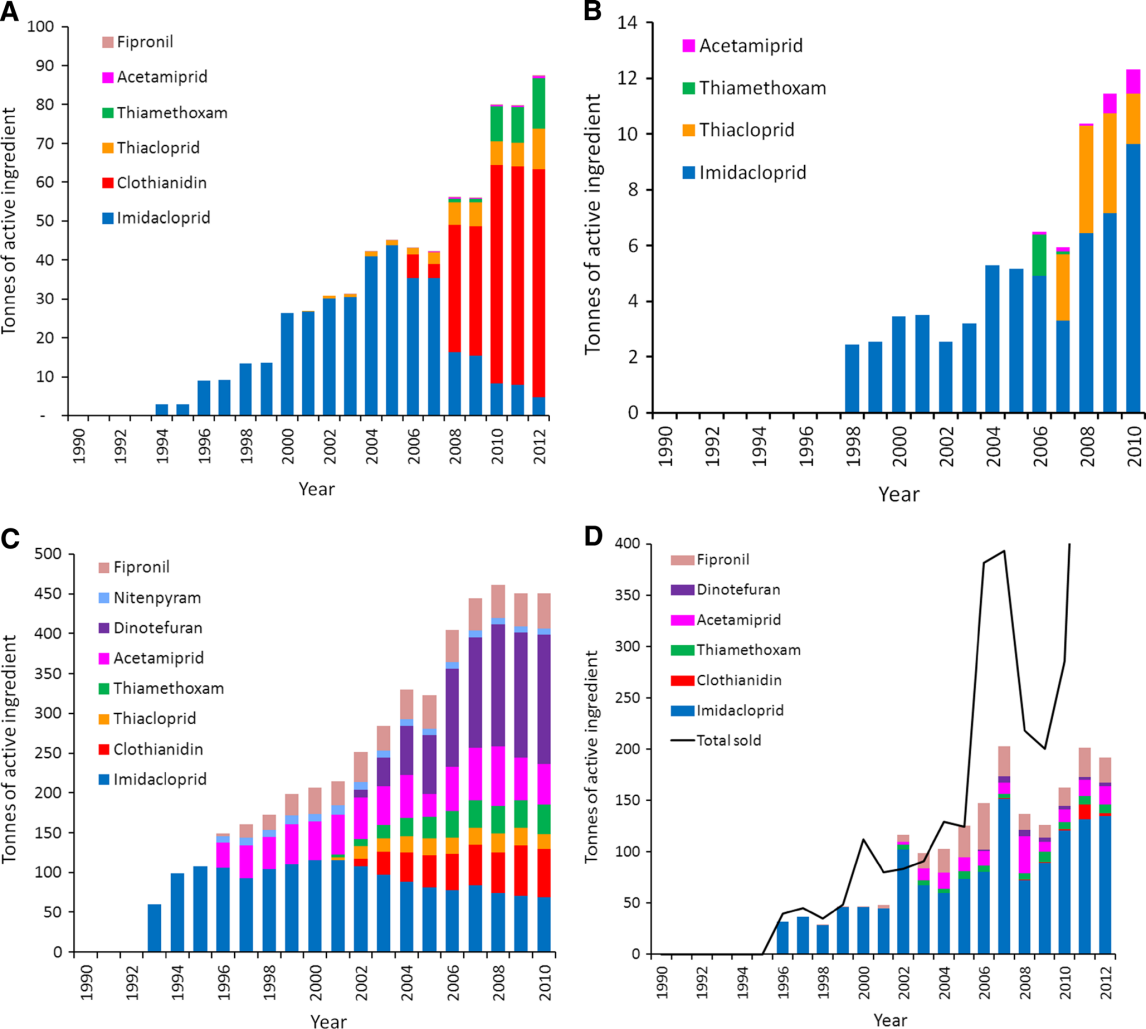
**a** Trend in the agricultural use of neonicotinoid insecticides in Britain from 1990, measured in tonnes of active ingredient applied per year. Data from http://pusstats.csl.gov.uk/index.cfm. **b** Trend in the quantities of neonicotinoid insecticides sold in Sweden from 1998, measured in tonnes of active ingredient per year. Data from Swedish Chemicals Agency, KEMI, quoted in (Bergkvist 2011). **c** Trend in the domestic shipment of neonicotinoid insecticides and fipronil in Japan from 1990, measured in tonnes of active ingredient per year. Data from Japan’s National Institute for Environmental Studies database, provided by Mizuno, R. in litt., 2012. **d** Trend in the quantity of neonicotinoid insecticides and fipronil used in California from 1990, measured in tonnes of active ingredient applied per year. Data taken from http://www.cdpr.ca.gov/docs/pur/purmain.htm. Also shown are the total quantities sold, from http://www.cdpr.ca.gov/docs/mill/nopdsold.htm

**Fig. 3:**
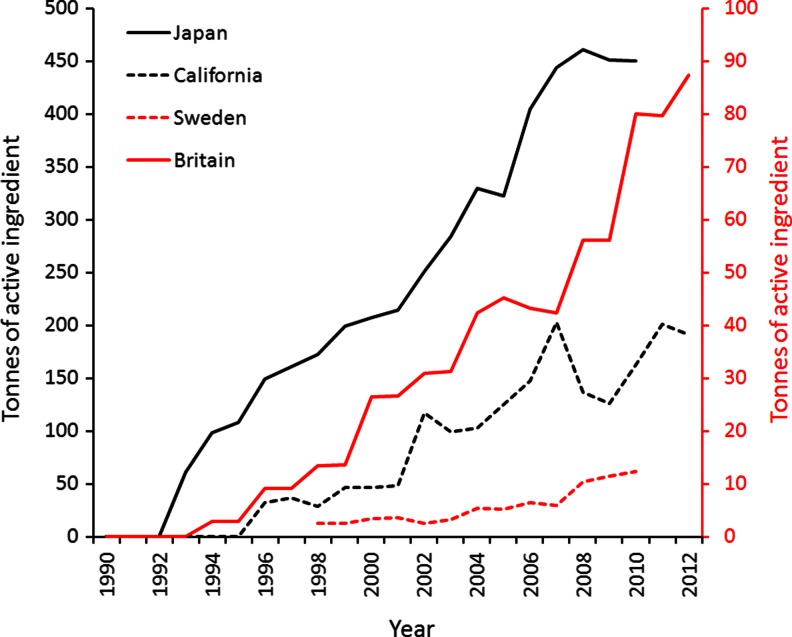
Trend in the sales (Sweden), domestic shipment (Japan), use (California) and agricultural use (Britain) of all neonicotinoid insecticides and fipronil. See Figs. 2a–d for further details. All measured in tonnes of active ingredient per year. Note the separate vertical axes for California//Japan, and Britain//Sweden

Obtaining country or state-specific information on annual trends in quantities used of neonicotinoid insecticides and fipronil is challenging. Such information is rare in the peer-reviewed literature. Furthermore, in those countries/states in which information is available (e.g., Great Britain, Sweden, Japan, and California), quantities are measured in different ways (sold, used, shipped, etc.) and comparisons of absolute amounts are not straightforward, though trends can be determined. For each of these countries and states, the overall use of neonicotinoids and fipronil has risen markedly since their first introduction in the early 1990s (Figs. 2a–d). There is little suggestion that the quantities sold, used, or shipped are reaching an asymptote (Fig. 3), which concords with the growth in their annual global sales (Table 1).

The quantities of neonicotinoid insecticides produced, sold, and applied may well continue to grow. This will be aided by the increases in the acreage of crops where they are heavily used, development of combined formulations (e.g., neonicotinoids combined with pyrethroids or fungicides), formulation technologies (e.g., Bayer CropScience’s Q-TEQ technology, which facilitates leaf penetration), the rise of generic (off-patent) products (Elbert et al. 2008; Jeschke et al. 2011), or possible development of molecules with properties of multiple pesticide classes (e.g., combinations of herbicidal and insecticidal properties).

Many insect pests have developed resistance to conventional insecticides such as organophosphates, carbamates, pyrethroids, chlorinated hydrocarbons, and insect growth regulators. Similarly, after nearly two decades of use, several target pests of neonicotinoids have begun to develop resistance (Jeschke et al. 2011). Examples are the greenhouse whitefly, *Trialeurodes vaporariorum* (Karatolos et al. 2010), the whitefly, *Bemisia tabaci* (Prabhakar et al. 1997; Cahill et al. 1996), and the Colorado potato beetle, *Leptinotarsa decemlineata* (Nauen and Denholm 2005; Szendrei et al. 2012; Alyokhin et al. 2007).

Wang et al. (2007) demonstrated a relationship between imidacloprid and acetamiprid resistance in cotton aphids (*Aphis gossypii*). An increase in the frequency of resistance to three neonicotinoids (acetamiprid, clothianidin, and thiamethoxam) has also been reported for *A. gossypii* by Herron and Wilson (2011). Shi et al. (2011) noted no cross-resistance between imidacloprid and two other neonicotinoids (thiamethoxam and clothianidin), but did find a 3.68–5.79-fold cross-resistance for acetamiprid, nitenpyram, and thiacloprid. These researchers concluded that resistance to acetamiprid and thiacloprid should be avoided on imidacloprid-resistant populations of *A. gossypii*.

Bioassays performed by Elbert and Nauen (2000) revealed a high degree of cross-resistance for the tobacco white fly (*B. tabaci*) to acetamiprid and thiamethoxam. Cross-resistance between imidacloprid and thiamethoxam was also confirmed under field conditions although Elbert and Nauen (2000) suggest that such problems are sometimes quite localized and that generalizations regarding resistance to imidacloprid or other neonicotinoids based on a few monitoring results should be avoided. Cross-resistance also appeared between imidacloprid, thiamethoxam, and clothianidin in the Colorado potato beetle, *L. decemlineata* (Alyokhin et al. 2007).

A recent study by Kavi et al. (2014) shows that resistance alleles to imidacloprid are present in the genetics of house flies (*Musca domestica*) in Florida. Imidacloprid selection resulted in a highly resistant strain of housefly, although the resistance was not stable and decreased over the course of several months. Incompletely dominant resistance of house flies to fipronil was found by Abbas et al. (2014).

The development of insecticide resistance against neonicotinoids in the brown planthopper (*Nilaparvata lugens*) was first observed in Thailand in 2003 and has since been found in other Asian countries such as Vietnam, China, and Japan. This problem has exacerbated yield losses in rice production in eastern China. Matsumura et al. (2008) found positive cross-resistance between imidacloprid and thiamethoxam in whitebacked planthopper, *Sogatella furcifera*, and also indicated that insecticide resistance of this crop pest against fipronil occurred widely in East and Southeast Asia. Planthopper resistance to imidacloprid has been reconfirmed following studies by Wang et al. (2008) and Azzam et al. (2011). According to Matsumura and Sanada-Morimura (2010) resistance to neonicotinoids is increasing. More recently, Zhang et al. (2014) studied nine field populations of the brown planthopper (*N. lugens*) from Central, East, and South China, and resistance to two neonicotinoids was monitored from 2009 to 2012. All nine field populations collected in 2012 had developed extremely high resistance to imidacloprid. Resistance to imidacloprid was much higher in 2012 than in 2009. Of the nine field populations, six populations showed higher resistance to nitenpyram in 2012 than in 2011.

Neonicotinoids are of enormous economic importance globally, especially in the control of pests that have previously developed resistance to other classes of insecticides (Jeschke et al. 2011). However, as for many pest control products, resistance to neonicotinoids may become a barrier to market growth if not managed appropriately. The systemic properties of neonicotinoid pesticides and fipronil, combined with prophylactic applications, create strong selection pressure on pest populations, thus expediting evolution of resistance and causing control failure. There is clearly a need to be judicious in our patterns of neonicotinoid use, given that the emergence of insecticide resistance can pose threats to crop production and food security.

## Uses

The use of neonicotinoids and fipronil covers four major domains: plant protection of crops and ornamentals against herbivorous insects and mites, urban pest control to target harmful organisms such as cockroaches, ants, termites, wasps, flies, etc., veterinary applications (against fleas, ticks, etc. on pets and cattle, and fleas in cattle stables) and fish farming (to control rice water weevil (*Lissorhoptrus oryzophilus* Kuscel) infestations in rice-crayfish (*Procambarus clarkii*) rotation (Barbee and Stout 2009; Chagnon et al. 2014)). Figures on the relative economic importance of these four domains of application are scarce, but to give an indicative example, the 2010 imidacloprid sales of Bayer CropScience (covering plant protection and biocide uses) amounted to 597 million Euro (Bayer CropScience 2011), while the 2010 imidacloprid sales of Bayer Healthcare (veterinary applications) amounted to 408 million Euro (Bayer Healthcare 2011). Overall, the largest use seems to be protection of crops, ornamentals, and trees in agriculture, horticulture, tree nursery, and forestry.

In agriculture, horticulture, tree nursery and forestry, neonicotinoids and fipronil can be applied in many different ways such as (foliar) spraying, seed dressing, seed pilling, soil treatment, granular application, dipping of seedlings, chemigation, (soil) drenching, furrow application, trunk injections in trees, mixing with irrigation water, drenching of flower bulbs and application with a brush on the stems of fruit trees. Seed and soil applications represent approx. 60 % of their uses worldwide (Jeschke et al. 2011). In Europe for instance, more than 200 plant protection products containing imidacloprid, thiamethoxam, clothianidin, acetamiprid, or thiacloprid are on the market. In 2012, these products had more than 1,000 allowed uses for the treatments of a wide range of crops and ornamentals including potato, rice, maize, sugar beets, cereals (incl. maize), oilseed rape, sunflower, fruit, vegetables, soy, ornamental plants, tree nursery, seeds for export, and cotton (EFSA 2012). In 2012, imidacloprid and thiamethoxam accounted for the largest share of authorized uses in Europe, with >30 and >25 %, respectively. Thiacloprid and acetamiprid accounted for >15 %, while clothianidin accounts for <5 %. These uses include field, greenhouse, and indoor applications. The largest share is field uses representing >60 % (EFSA 2012). Approximately 70 % of the number of allowed field uses in Europe were spray applications in 2012, while less than 20 % were seed treatment and less than 20 % were other methods of application such as drip irrigation, soil treatment. However, it is worthwhile noting here that “percentage of number of allowed uses” is not the same as “percentage of the total volume of active substance,” nor is it representative of the extent of treated area. Thiacloprid and acetamiprid are authorized in the EU as spray or soil treatments. In Europe, no uses as seed treatment were noted for acetamiprid, and a single use was noted for thiacloprid (maize) (EFSA 2012). In Asia, major large-scale applications of neonicotinoids include spraying of rice fields and other crops (Taniguchi et al. 2012), as well as granular applications (Thuyet et al. 2011, 2012) and seed coatings.

**Fig. 4:**
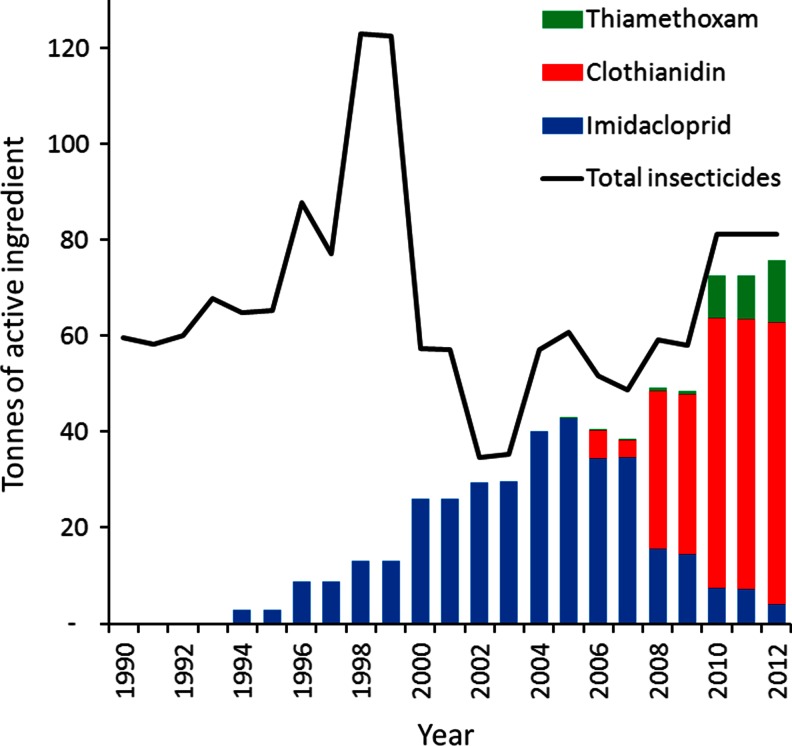
Trend in the agricultural use of neonicotinoid insecticides as seed treatments in Britain from 1990, measured in tonnes of active ingredient per year (*bars*). The total usage of all insecticidal seed treaments (*solid line*) is also shown. Data from http://pusstats.csl.gov.uk/index.cfm

By far, the largest and most popular application in crop protection is the prophylactic seed coating. It is an a priori treatment against target pests that may decrease production yields. During germination and growing, the active substance in the seed coating is taken up by the roots and translocated to all parts of the crop, making the crop toxic to insects that attempt to feed upon it (Van der Sluijs et al. 2013). The global market for coating crop seeds with insecticides grew dramatically (more than six-fold) between 1990 and 2008, when its total value approached a billion Euros (Jeschke et al. 2011). This growth was almost entirely due to seeds being treated with neonicotinoids, which are well suited to this form of application (Elbert et al. 2008). In Britain, for example, of the 87.2 tonnes of neonicotinoid applied in 2012, 75.6 tonnes was as a seed treatment. In fact, 93 % by weight of all insecticidal seed treatment was with neonicotinoids (Fig. 4).

Similarly, the largest use of these compounds in North America is via application to seed in many annual row crop systems. Corn (maize) is the largest single use—in fact, production of corn for food, feed, and bioethanol production represents the largest single use of arable land in North America. Pest management of seed and seedling disease and insect pests in corn is achieved almost exclusively using prophylactic applications of pesticide “cocktails” that routinely include neonicotinoid seed treatments for insect control. One coated maize seed typically is coated with between 1,500 and 4,500 ppm of insecticide (or 0.5–1.5 mg per seed). Systemic and long-lasting high concentrations allow not only the protection of the seedling from soil-bound insects but also offer some suppression of western corn rootworm, *Diabrotica virgifera virgifera*, whose attacks usually start one or more weeks after the sowing (van Rozen and Ester 2010).

Maize planting reached unprecedented levels in the USA in 2013 at 96 million acres, or 38.8 million ha (USDA-NASS 2013). This level of production is expected to increase in 2014 and beyond. Virtually all of the seeds planted in North America (the lone exception being organic production = 0.2 % of total acreage, USDA –NASS 2013) are coated with neonicotinoid insecticides. The two major compounds used are clothianidin and thiamethoxam; the latter is metabolized to clothianidin in insects, other animals, plants, and soil (Nauen et al. 2003). Although maize is the largest single use, seed treatments in other large acreage crops, including soybeans (31.4 million ha), wheat (23 million ha), and cotton (4.2 million ha) combine to make this class of insecticides the most widely used in the USA in history, when measured by area of application (USDA-NASS 2013).

Neonicotinoid seed treatments are routinely applied to the vast majority of grain and oilseed crops in developed countries, regardless of pest pressures or field histories. Untreated seeds are often unavailable for purchase. In fact, in many of the most important crops grown in North America (notably maize), there are no non-neonicotinoid seed alternatives readily available to producers in the marketplace. Because any subsequent crop insurance claims by producers must document that accepted standard practices were used during planting, there is an inherent risk in requesting seed that is markedly different from the standard. This may present a disincentive for producers that would otherwise attempt growing untreated seeds in some fields. Several efficacy studies have demonstrated that applications of neonicotinoids can reduce pest population densities, defoliation, and crop damage (e.g., Maienfisch et al. 2001b; Kuhar et al. 2002; Nault et al. 2004; Koch et al. 2005). This can result in increased crop yields compared to crops with no pest management (see review by Jeschke et al. 2013).

However, because the pests targeted by neonicotinoids are generally occasional, sporadic, and secondary pests, these benefits are not routinely found: a review of literature by Stevens and Jenkins (2014) found inconsistent benefits in 11 of 19 peer-reviewed papers examined, and no benefit in the remaining 8 articles. Considering the nature of the pests targeted, this is not altogether surprising. By definition, these secondary pests are often not present or present in subeconomic levels. However, they do occur and it is crucial that crop producers have options for management. These resources do exist: there is a significant base of knowledge for managing these secondary pests, and agricultural practices such as crop rotation drastically reduce the need for control through neonicotinoids in many cases (Apenet 2009, 2010, 2011). Indeed, the cost-effectiveness of the prophylactic use of neonicotinoids has in the past and recently been questioned (Maini et al. 2010; Stevens and Jenkins 2014). Several studies have shown that the use of neonicotinoids does not necessarily result in increased yield or economic benefit, thereby bringing into question the advisability of a widespread and prophylactic use of neonicotinoid insecticides (Apenet 2011; Mole et al. 2013; Stokstad 2013). Macfadyen et al. (2014) showed that imidacloprid-treated seeds tended to increase yields of canola, but no such benefit was found for wheat. Similarly, Royer et al. (2005) found that imidacloprid-treated seeds sometimes increased yields of wheat but did not always result in a positive economic return. Neonicotinoid insecticidal seed treatments provided no yield benefits over a 2-year study in experimental soybean applications (Seagraves and Lundgren 2012). De Freitas Bueno et al. (2011) also found that the prophylactic use of neonicotinoids on soybeans did not significantly increase production in comparison to other pest management approaches. Johnson et al. (2009) found that although imidacloprid treatments increased the yield of soybeans, the economic return from imidacloprid-treated crops was not as high as those from crops under an integrated pest management program. In citrus orchards of California, imidacloprid treatments were ineffective or marginally effective at controlling damage from scales or mites and the insecticides suppressed natural enemies such that overall benefits to citrus crops were less than from other pest management options including growth regulators (Grafton-Cardwell et al. 2008). Taken as a whole, these data reflect that use levels for neonicotinoid seed treatments are dramatically out of step with the actual need; in most cases, pests are absent or present at such low numbers that seed treatments cannot demonstrate any benefit.

Alternatives to this prophylactic use of neonicotinoids including those presented by Furlan and Kreutzweiser (2014) may help to minimize the risk of insect and other arthropod resistance (see above) to neonicotinoids and reduce overall operational costs.

## Mode of action on invertebrates

Neonicotinoids can be considered substances acting as agonists on nAChRs opening cation channels (Casida and Durkin 2013). Voltage-gated calcium channels are also involved (Jepson et al. 2006) in their insecticidal activity (Liu et al. 1995; Orr et al. 1997; Nishimura et al. 1998; Tomizawa and Casida 2001, 2003, 2005). Differences in properties and structure of the subunits between insects and mammalian nAChRs explain in part the high selectivity of neonicotinoids to arthropods and the supposed relatively low toxicity to vertebrates (Nauen et al. 1999; Lansdell and Millar 2000; Matsuda et al. 2001; Tomizawa and Casida 2003, 2005). Electrophysiological studies have shown that the binding potency of neonicotinoids to brain membranes is well and positively correlated with their agonistic and insecticidal activity. This suggests that the channel opening of nAChRs induced by the binding of neonicotinoids to receptors leads to insecticidal activity (Nishimura et al. 1998; Nishiwaki et al. 2003). As a result, their agonistic action induces continuous excitation of the neuronal membranes, producing discharges leading to paralyses and cell energy exhaustion. This binding potency is conferred by a unique molecular conformation (Tomizawa and Casida 2011). However, the interaction of this conformation with the receptor may vary depending on their different chemical substituents and on the species considered (Honda et al. 2006). In addition, the sensitivity of insect nAChRs to neonicotinoids may be modulated by phosphorylation mechanisms, as shown for imidacloprid (Salgado and Saar 2004), leading to variation in the insecticidal activity. Thus, imidacloprid selectively inhibits desensitizing nicotinic currents, while displaying a selective desensitization toward certain nAChR subtypes (Oliveira et al. 2011). This indicates that selective desensitization of certain nAChR subtypes can account for the insecticidal actions of imidacloprid.

The characterization of the binding sites, the recognition subsites, and the toxicophores of neonicotinoids have been studied in depth (Hasegawa et al. 1999; Kagabu et al. 2002; Kanne et al. 2005; Matsuda et al. 2005; Kagabu 2008; Kagabu et al. 2008; Kagabu et al. 2009). Photoaffinity labelling has enabled identification of the amino acids involved in the molecular interaction between neonicotinoids and nAChRs or the acetylcholine binding protein (AChBP) (Tomizawa and Casida 1997; Kagabu et al. 2000; Tomizawa et al. 2001a; Tomizawa et al. 2001b; Zhang et al. 2002, 2003; Tomizawa et al. 2007; Tomizawa et al. 2008; Tomizawa and Casida 2009). It appears that, in the same binding pocket, two very different interactions drive the recognition of neonicotinoids. The electronegative toxicophore of neonicotinoids and the cationic toxicophore of nicotinoids (nicotine, epibatidine, and desnitro-imidacloprid) lead to them docking in opposite directions at the binding sites (Tomizawa et al. 2003; Tomizawa and Casida 2009).

Neonicotinoids appear to bind to multiple sites on membranes of neural tissues in various insect species. The American cockroach, *Periplaneta americana*, expresses two types of receptors resistant to α-bungarotoxin (α-BgTx), an antagonist of nicotinic receptors: nAChR1, which is sensitive to imidacloprid, and nAChR2, which is not (Courjaret and Lapied 2001; Courjaret et al. 2003; Tan et al. 2007; Thany et al. 2008). As a result, while imidacloprid acts on nAChR1 and not on nAChR2, nicotine, acetamiprid, and clothianidin act as agonists of nAChR2 (Bordereau-Dubois et al. 2012; Calas-List et al. 2013).

The first generation of neonicotinoids included nitenpyram, imidacloprid, acetamiprid, and thiacloprid. Imidacloprid and its metabolites are highly toxic to bees (Suchail et al. 2000, 2001). It behaves like a partial agonist of the nicotinic nAChRs in Kenyon cells of the honey bee (*Apis mellifera*) mushroom body, which are involved in higher order neuronal processes in the brain such as olfactory learning (Déglise et al. 2002). However, the pharmacological properties and the molecular composition of nAChRs differ in Kenyon cells and in neurons from antennal lobes (Barbara et al. 2008; Dupuis et al. 2011). In antennal lobe neurons, the characterization of type I nAChR currents, which exhibit slow desensitization, and type II currents, which exhibit fast desensitization, strongly suggest the presence of at least two different types of nAChRs. The presence of two types of receptors displaying different affinities for imidacloprid and its metabolites was proposed on the basis of the complex toxicity profile after acute and chronic exposures in the honey bee (Suchail et al. 2001). Such complex profiles can be shown both on mortality rates and on sublethal effects on reproduction. This has been recently exemplified for common fruit fly, *Drosophila melanogaster*, after chronic exposure to imidacloprid, at concentrations far below the levels found in the field (Charpentier et al. 2014). A study designed to demonstrate the absence of different biological targets of imidacloprid and its metabolites (Nauen et al. 2001) proved inconclusive for several reasons: (1) a binding of [^3^H]-imidacloprid occurs at nanomolar concentrations, whereas ionic currents are induced at micromolar concentrations (30 μM here), (2) the pharmacology of the current induced by imidacloprid, 5-OH-imidacloprid and olefin (two important metabolites of imidacloprid, see metabolites section for details) has not been investigated, (3) no Scatchard analysis is presented, therefore no analysis for receptor binding interactions is provided, and (4) displacement experiments have been performed at nanomolar concentrations instead of micromolar concentrations, which prevent the dual characterization of high and low-affinity targets. Studies on the effects of imidacloprid and two of its metabolites, 5-OH-imidacloprid and olefin-imidacloprid, on the habituation phenomenon have enabled the characterization of two receptors differentially expressed during honey bee development (Guez et al. 2001; Guez et al. 2003).

The occurrence of two types of imidacloprid targets, which could explain the differential toxicity of imidacloprid at low and very low doses observed in bees, has been demonstrated in the green peach aphid (*Myzus persicae*). Saturable binding of [^3^H]-imidacloprid has revealed a high-affinity binding site, with a dissociation constant (*K*_d_) of 0.14 nM, and a low-affinity binding site, with *K*_d_ of 12.6 nM, whose pharmacology resembles that of nAChR (Lind et al. 1998). Another study confirming these results presented similar dissociation constants of 0.6 and 7.2 nM (Wiesner and Kayser 2000). In addition, the pharmacology of the high-affinity binding site is similar to that of α-BgTx binding sites in the honey bee and the hawk moth (*Manduca sexta*) (Lind et al. 1999). The existence of two imidacloprid binding sites has been confirmed in the brown planthopper (*N. lugens*) (Li et al. 2010). Two [^3^H]-imidacloprid binding sites have been identified with different affinities (*K*_d_ = 3.5 pM and *K*_d_ = 1.5 nM) and subunit co-assemblies (α1, α2, and β1 for the low-affinity nAChR and α3, α8, and β1 for the high-affinity nAChR). In fact, the existence of multiple binding sites in insects seems to appear as a relatively common feature of neonicotinoids, since it has also been observed in the aphid (*Aphis craccivora*) and in the locust (*Locusta migratoria*) (Wiesner and Kayser 2000).

Contrary to acetylcholine, acetylcholinesterase does not act on nicotine nor imidacloprid, and possibly on the other neonicotinoids, leading to their prolonged action on the nAChRs (Thany 2010). Furthermore, poor neuronal detoxification mechanisms may contribute to a prolonged action at this level (Casida and Durkin 2013). 6-chloronicotinic acid (6-CNA) is a metabolite common to chloropyridinyl neonicotinoids (Ford and Casida 2008; Casida 2011). Some of these metabolites have proved to be highly toxic to bees leading to significant mortalities by chronic exposure (Suchail et al. 2001). Thus, the risk posed by 6-CNA to the honey bee might be common to the use of imidacloprid, thiacloprid, acetamiprid, and nitenpyram. These features may contribute to the delayed and chronic lethality observed with some neonicotinoids, e.g., thiacloprid, imidacloprid (Suchail et al. 2001; Beketov and Liess 2008; Tennekes and Sánchez-Bayo 2011; Roessink et al. 2013).

Imidacloprid has been shown to stimulate plant growth of genetically modified stress tolerant plants, even in the absence of damaging pest species, leading to increase in crop yield. As a result, treated plants respond better to the effects of abiotic stressors such as drought (Thielert et al. 2006). The metabolite 6-CNA has been suggested to be responsible for the physiological plant changes as it is known to induce a plant’s own defenses against plant disease. Consequently, imidacloprid together with acetamiprid, thiacloprid, and nitenpyram are included within the so-called Stress Shield^TM^ technology (Bayer 2006).

Thiamethoxam, a second-generation neonicotinoid (Maienfisch et al. 2001a), acts differently to first-generation neonicotinoids. Thiamethoxam is a poor agonist of insect nAChRs (Nauen et al. 2003; Tan et al. 2007; Benzidane et al. 2010). However, it is a full agonist at cercal afferent/giant interneuron synapses (Thany 2011) where it induces a strong depolarization that can be partially lowered by the muscarinic antagonist atropine. This suggests that thiamethoxam is able to bind to mixed nicotinic/muscarinic receptors (Lapied et al. 1990). Metabolic N-desmethylation of thiamethoxam (TMX-dm) results in an increase in the affinity to the [^3^H]-imidacloprid binding site (Wiesner and Kayser 2000). However, although it does not occur in lepidopteran larvae, TMX-dm can be produced in mammals and insects (Nauen et al. 2003; Ford and Casida 2006b). It can interact with insect nAChRs, but is about 25 times less potent than thiamethoxam as an insecticide (Nauen et al. 2003), but is nevertheless marketed in its own right. The thiamethoxam metabolite, clothianidin, presents insecticidal activity (Nauen et al. 2003). It can act on imidacloprid-sensitive nAChR1 and imidacloprid-insensitive nAChR2 subtypes (Thany 2009, 2011). Studies involving neurophysiology, behavioral experiments, and chemical analysis have revealed that the effect of thiamethoxam on cockroach locomotor activity is closely associated with the appearance of its metabolite clothianidin (Benzidane et al. 2010). These two molecules are often presented together in environmental matrices (Bonmatin et al. 2014), and their toxic action may therefore be enhanced.

The third-generation neonicotinoid dinotefuran (Wakita et al. 2003) can interact with insect nAChRs (Mori et al. 2002; Kiriyama et al. 2003). A high-affinity binding site, exhibiting a dissociation constant of 13.7 nM, has been characterized in the nerve cord membranes of the American cockroach (*P. americana*) (Miyagi et al. 2006). However, Scatchard analysis suggests the occurrence of two binding sites. Dinotefuran can exhibit a nerve-excitatory activity, which is lower than that of imidacloprid and comparable to that of clothianidin, and a nerve-blocking activity, which is comparable to that of imidacloprid and slightly higher than that of clothianidin (Kiriyama and Nishimura 2002). Such a nerve-blocking action has also been described in cockroaches with thiacloprid and its derivatives (Kagabu et al. 2008). The insecticidal activity of dinotefuran and its derivatives is better correlated to nerve-blocking activity than to nerve-excitatory activity, a characteristic also observed with other neonicotinoids (Kagabu et al. 2008). Both the nitroguanidine and the terahydro-3-furylmethyl parts of the molecule are important for the insecticidal activity of dinotefuran (Wakita et al. 2004a; Wakita et al. 2004b; Wakita 2010). However, compared to imidacloprid and acetamiprid, dinotefuran appears more effective in inducing depolarizing currents in terms of current amplitude and concentration dependence (Le Questel et al. 2011).

Sulfoxaflor is a fourth-generation neonicotinoid that exhibits a high insecticidal activity against a broad range of sap-feeding insects (Babcock et al. 2011). It can also act on nAChRs and may be considered as a neonicotinoid. This needs to be taken into account when considering possibilities for insecticide rotation in order to manage resistance toward neonicotinoids (Cutler et al. 2013). The nature of the interactions with nAChRs differs between sulfoxaflor and the other neonicotinoids (Sparks et al. 2013). Sulfoxaflor induces currents of high amplitude when tested on nAChR hybrids of *D. melanogaster* α2 nAChR subunit and chicken β2 subunit in the african clawed frog (*Xenopus laevis*) oocytes (Watson et al. 2011). The maximum intensity (*I*_max_) of sulfoxaflor-induced currents is much higher than those of imidacloprid, acetamiprid, thiacloprid, dinotefuran, and nitenpyram. Conversely, sulfoxaflor presents a weak affinity to displace [^3^H]-imidacloprid from green peach aphid (*M. persicae*) membranes. In stick insect (Phasmatodea) neurons, sulfoxaflor potently desensitizes fast-desensitizing currents, *I*_ACh1H,_ and both slowly desensitizing components, *I*_ACh2H_ and *I*_ACh2L_ (Oliveira et al. 2011). These studies clearly show that the action of sulfoxaflor and other sulfoximines, similar to that of imidacloprid, involves receptor desensitization, receptor selectivity, a differential action at low and high doses and, probably, receptor desensitization after a prolonged exposure. Additionally, the use of *D. melanogaster* strains presenting mutations at Dα1 and Dβ2 nAChR subunits, or resistant silverleaf whitefly (*B. tabaci*) strains revealed no cross-resistance between sulfoxaflor and imidacloprid or spinosyns (family of compounds with insecticidal activity produced from fermentation of two species of *Saccharopolyspora*, including active ingredients such as spinosad; Perry et al. 2012; Longhurst et al. 2013), despite the fact that sulfoxaflor shares nAChR as a common target with other neonicotinoids.

The pharmacology of cycloxaprid, a *cis*-neonicotinoid also belonging to the fourth generation, has been subjected to fewer investigations due to its recent discovery. In the housefly, [^3^H]-cycloxaprid binds to head membranes with a *K*_d_ of 28 nM (Shao et al. 2013b). Displacement studies show that the cycloxaprid metabolite, [^3^H]-nitromethylene imidazole (NMI), is 19, 15, and 41-fold more potent than cycloxaprid on housefly, honey bee, and mouse (*Mus musculus*) brain membranes, respectively.

Neonicotinoids induce depolarizing currents in insects by an agonist action on nAChRs. However, as seen above, they also exert a nerve-blocking activity that contrasts with their agonist action and their nerve-excitatory activity, as shown for thiacloprid and its derivatives (Kagabu et al. 2008; Toshima et al. 2008). Studies carried out at chicken neuromuscular junction strongly suggest that imidacloprid is an antagonist at muscle cell nAChRs (Seifert and Stollberg 2005). In *N. lugens*, the Y151S mutation in Nlα1 subunit is associated with a resistance to imidacloprid, but has little effect on the action of acetylcholine (Liu et al. 2005; Liu et al. 2006). Replacement of tyrosine with methionine (Y151M mutation), as found in *Caenorhabditis elegans* in the site equivalent to Y151, instead of serine, results in Nlα1/β2 nAChR on which imidacloprid acts as an antagonist (Zhang et al. 2008). This shows that very subtle differences in subunit sequence can lead to nAChRs resistant to neonicotinoids or to nAChRs on which neonicotinoids can act agonistically or antagonistically.

As with carbamates and organophosphates, fipronil exerts its insecticidal activity by acting on the inhibiting system of the nervous system. It binds to GABA receptors (Tingle et al. 2003) and to glutamate receptors coupled to chloride channels (Barbara et al. 2005). In doing so, fipronil blocks the inhibiting receptors leading to an excitation of the nervous system. It leads to neuronal hyperexcitation due to accumulation of the neurotransmitter (GABA) at the synaptic junctions. Its mode of action is, therefore, antagonistic, whereas that of neonicotinoids is agonistic. Glutamate receptors are insect specific, which is the reason why fipronil is more effective on invertebrates than on vertebrates (Narahashi et al. 2007). Furthermore, it seems to have low affinity to vertebrate receptors (Grant et al. 1998). Fipronil shows a higher selectivity for insects than for humans, with affinity constant (*K*_*I*_ = IC50 / (1 + [*L*] / *K*_d_)) of 4 nM for the housefly GABA_A_ receptors and 2,169 nM for human GABA_A_ receptors (Ratra and Casida 2001). However, selectivity and sensitivity may vary with the subunit composition of the human GABA_A_ receptors. Competition with the binding of 4-[^3^H]-ethylnylbicycloorthobenzoate ([^3^H]-EBOB) to GABA receptors was performed to compare the relative affinity of fipronil to GABA receptors of different subunit compositions (Ratra et al. 2001). Fipronil is highly selective to the β3 receptors (*inhibitory concentration 50 %* (*IC*_50_) = 2.4 ± 0.3 nM; *K*_*I*_ = 1.8 nM), but presents a lower selectivity to native GABA_A_ receptors (*IC*_50_ = 2,470 ± 370 nM; *K*_*I*_ = 2,160 nM). The fact that native receptors show a lesser affinity to fipronil than β3 receptors suggests that the other subunits of the human GABA_A_ receptors modulate the sensitivity of GABA receptors to fipronil (Casida and Quistad 2004). Fipronil derivatives show a higher affinity for native receptors than fipronil, with *IC*_50_ values ranging between 237 ± 45 and 343 ± 49 nM for the derivatives, and 2,470 ± 370 nM for fipronil (Ratra et al. 2001). Fipronil interacts with AChR receptors with lower affinity than neonicotinoids (Barbara et al. 2005).

## Metabolites

Metabolism of the seven major commercial neonicotinoids can be divided into two phases. Phase I metabolism, largely dependent on cytochrome P450, includes reactions such as demethylation, nitro reduction, cyano hydrolysis, hydroxylation of imidazolidine and thiazolidine accompanied by olefin formation, hydroxylation of oxadiazine accompanied by ring opening, and chloropyridinyl and chlorothiazolyl dechlorination (Ford and Casida 2008; Casida 2011). For some neonicotinoids, cytosolic aldehyde oxidase together with cytochrome P450 is responsible for nitro reduction in mammals (Dick et al. 2005; Casida 2011). Phase I metabolites have been found in both small mammals and plants (Chen et al. 2005; Casida 2011). Phase II metabolism is mainly responsible for conjugate formation, which differ between plants and animals (Ford and Casida 2008; Casida 2011). Several metabolites are common to different neonicotinoids but others are compound specific (Schulz-Jander and Casida 2002; Ford and Casida 2006a; Ford and Casida 2008; Shi et al. 2009; Casida 2011).

Neonicotinoids are subjected to intense metabolism in plants leading to the appearance of different metabolites during the plant life or, at least, up to the harvest of plants consumed by humans or breeding animals (Laurent and Rathahao 2003; Greatti et al. 2006; Ford and Casida 2008; Karmakar et al. 2009; Karmakar and Kulshrestha 2009). As a result, metabolites may induce a long-lasting action of neonicotinoids against pests, particularly plant-sucking pests such as aphids (Nauen et al. 1998). Tables 2 and 3 show the metabolites of neonicotinoids and fipronil, respectively.

**Table 2:** Metabolites of neonicotinoids in various media and organisms. Metabolites known to be active toward invertebrates or mammals are highlighted in bold

Parent compound	Metabolites	Formation medium	Reference
Thiamethoxam (TMX)	**Clothianidin, CLO**	Soil, mice, mammals, insects, plants	(Ford and Casida 2006a); Nauen etl al. 2003; PPDB 2013; FAO thiamethoxam
	**Thiamethoxam-dm, TMX-dm,** **N-desmethyl thiamethoxam**	Mice	(Ford and Casida 2006a)
	**TMX-NNO**	Mice, soil bacteria (*Pseudomonas* sp.)	(Ford and Casida 2006a) (Pandey et al. 2009)
	**TMX-NNH2**	Mice	(Ford and Casida 2006a)
	**TMX-NH**	Mice, soil bacteria (*Pseudomonas* sp.), water (photodegradation), soil	(Ford and Casida 2006a); (Pandey et al. 2009); (De Uderzo et al. 2007); FAO thiamethoxam
	TMX-Urea	Mice, soil bacteria (*Pseudomonas* sp.), water (photodegradation), soil	(Ford and Casida 2006a); (Pandey et al. 2009); (Schwartz et al. 2000); FAO thiamethoxam
	**TMX-dm-NNO**	Mice	(Ford and Casida 2006a)
	**TMX-dm-NH2**	Mice	(Ford and Casida 2006a)
	**TMX-dm-NH**	Mice	(Ford and Casida 2006a)
	TMX-dm-Urea	Mice	(Ford and Casida 2006a)
	hydroxy thiazole urea derivative	Plants (tomato)	(Karmakar et al. 2009)
	6-hydroxy oxadiazinon	Plants (tomato)	(Karmakar et al. 2009)
	ether derivative	Plants (tomato)	(Karmakar et al. 2009)
	NG-A	Mammals	(Ford and Casida 2006a)
	NG-B	Mammals	(Ford and Casida 2006a)
	NG-C	Mammals	(Ford and Casida 2006a)
	NG-D	Mammals	(Ford and Casida 2006a)
	5-methyl-2(3H)-thiazolone	Water (photodegradation)	(De Uderzo et al. 2007)
	oxazine derivative	Water (photodegradation)	(De Uderzo et al. 2007)
	acrylonitrile derivative	Water (photodegradation)	(De Uderzo et al. 2007)
	carbonyl sulfide	Water (photodegradation)	(De Uderzo et al. 2007); (Schwartz et al. 2000)
	isocyanic acid	Water (photodegradation)	(De Uderzo et al. 2007); (Schwartz et al. 2000)
Clothianidin/Thiamethoxam	**TZNG, CLO-dm** **N-(2-chlorothiazol-5- ylmethyl)-N′-nitroguanidine**	Soil, plants, mammals	PPDB 2013; (Kim et al. 2012); (Ford and Casida 2006a), FAO clothianidin; (Ford and Casida 2008)
	**CLO-NNO**	Mice, insects, plants	(Ford and Casida 2006a); (Kanne et al. 2005); (Ford and Casida 2008), (Karmakar et al. 2009)
	**CLO-dm-NNO**	Mice, insects, plants	(Ford and Casida 2006a); (Kanne et al. 2005); (Ford and Casida 2008)
	**CLO-NNH2, ATMG**	Mice, insects	(Ford and Casida 2006a); (Kanne et al. 2005)
	**CLO-dm-NNH2, ATG**	Mice, insects	(Ford and Casida 2006a); (Kanne et al. 2005)
	**CLO-NH, TMG, N-(2-chlorothiazol-5-ylmethyl)-N′-methylguanidine**	Soil, plants, sediment, mammals	(Kim et al. 2012); (Ford and Casida 2006a); FAO clothianidin; (Ford and Casida 2008)
	CLO-dm-NH, TZG	Mammals, plants	(Ford and Casida 2006a); FAO clothianidin; (Ford and Casida 2008)
	**CLO-Urea, TZMU, N-(2-chlorothiazol-5-ylmethyl)-N-methylurea**	Soil, Plants, mammals, water	PPDB 2013; (Kim et al. 2012); FAO clothianidin; (Ford and Casida 2008); (Karmakar et al. 2009); Žabar et al. 2012; (Schwartz et al. 2000)
	**CLO-dm-Urea, TZU, 2-chloro-1,3-thiazole-5-ylmethylurea**	Mammals, plants, soil	(Kim et al. 2012); (Ford and Casida 2006a); FAO clothianidin; (Ford and Casida 2008)
	THMN, N-hydroxy clothianidin, N-2-Chlorothiazol-5-ylmethyl-N-hydroxy-N′-methyl-N″-nitroguanidine	Rat, apple	FAO clothianidin
	2-chloro-1,3-thiazole-5-methylamine	Tomato cell culture	(Karmakar et al. 2009)
	2-chloro-1,3-thiazole-5-methyl isocyanate	Tomato cell culture	(Karmakar et al. 2009)
	TZA, CTM-a	Mammals	(Ford and Casida 2006a)
	TZOH, CTM-b*	Mammals	(Ford and Casida 2006a)
	CTM-c, CTA, CTCA, 2-chloro-1,3-thiazole-5-carboxylic acid	Mammals, plants	(Kim et al. 2012); (Ford et al. 2010); (Ford and Casida 2008); (Ford and Casida 2006a)
	CTM-i, cACT, 2-chlorothiazol-5-ylmethylamine	Water	FAO clothianidin
	CTM-f	Mammals	(Ford and Casida 2006a)
	CTNU, N-(2-chlorothiazol-5-ylmethyl)-N′-nitrourea	Water	FAO clothianidin
	HMIO, 4-hydroxy-2-methylamino-2-imidazolin-5-one	Water	FAO clothianidin
	MIT, 7-methylamino-4H-imidazo[5,1-b][1,2,5]thiadiazin-4-one	Water	FAO clothianidin
	FA, Formamide	Water	FAO clothianidin
	MU, Methylurea	Water	FAO clothianidin
Thiamethoxam/Clothianidin/Dinotefuran	MNG, NG-E, N-methyl-N-nitroguanidine	Soil, plants, mammals	PPDB 2013; (Ford and Casida 2006a, b); FAO clothianidin
Thiamethoxam/Clothianidin/Dinotefuran	MG, NG-F, Methylguanidine	Water, plants, mammals	(Kim et al. 2012); (Ford and Casida 2006a); FAO clothianidin
Thiamethoxam/Clothianidin/Dinotefuran	NG-G, NTG, nitroguanidine	Mammals, soil, plants	(Ford and Casida 2006a); FAO clothianidin
Dinotefuran	DIN-dm, FNG, N-desmethyl dinotefuran, 2-nitro-1-(tetrahydro-3-furylmethyl)guanidine	Mammals, plants, soil (aerobic)	(Ford and Casida 2006a); (Ford and Casida 2008); FAO dinotefuran
	DIN-NNO	Mammals, plants	(Ford and Casida 2006a); (Ford and Casida 2008)
	DIN-dm-NNO	Mammals, plants	(Ford and Casida 2006a); (Ford and Casida 2008)
	DIN-NNH2	Mammals, plants	(Ford and Casida 2006a); (Ford and Casida 2008)
	DIN-dm-NNH2	Mammals, plants	(Ford and Casida 2006a); (Ford and Casida 2008)
	**DIN-NH, DN, 1-Methyl-3-(tetrahydro-3-furylmethyl)guanidine**	Mammals, plants, water (photolysis), soil (anaerobic)	(Ford and Casida 2006a); (Ford and Casida 2008); FAO dinotefuran; USEPA 2004b
	**DIN-dm-NH, 3-(tetrahydro-3-furylmethyl) guanidine**	Mammals, plants	(Ford and Casida 2006a); (Ford and Casida 2008)
	**DIN-Urea, UF, 1-Methyl-3-(tetrahydro-3-furylmethyl)urea**	Mammals, plants, soil (aerobic), water (hydrolysis + photolysis)	(Ford and Casida 2006a); (Ford and Casida 2008); (Rahman et al. 2013); FAO dinotefuran; USEPA 2004b
	**DIN-dm-Urea, 3-(tetrahydro-3-furylmethyl)urea**	Mammals, plants	(Ford and Casida 2006a); (Ford and Casida 2008)
	**DIN-2-OH**	Mammals, plants, water (photolysis)	(Ford and Casida 2006a); FAO dinotefuran; USEPA 2004b
	**DIN-5-OH**	Mammals, plants	(Ford and Casida 2006a); (Ford and Casida 2008)
	DIN-4-OH	Mammals	(Ford and Casida 2006a)
	DIN-a, PHP, 1,3-diazinane aminocarbinol (derivative of DIN-2OH)	Mammals, plants	(Ford and Casida 2006a); FAO dinotefuran
	DIN-b (derivative of DIN-dm)	Mammals	(Ford and Casida 2006a)
	DIN-e (guanidine derivative of DIN-a)	Mammals	(Ford and Casida 2006a)
	DIN-f (guanidine derivative fo DIN-b)	Mammals	(Ford and Casida 2006a)
	DIN-g (derivative of DIN-5-OH)	Mammals, plants	(Ford and Casida 2006a); (Ford and Casida 2008)
	DIN-h (desmethyl DIN-g)	Mammals, plants	(Ford and Casida 2006a); (Ford and Casida 2008)
	DIN-i (nitroso derivative of DIN-g)	Mammals, plants	(Ford and Casida 2006a); (Ford and Casida 2008)
	DIN-j (nitroso derivative fo DIN-h)	Mammals, plants	(Ford and Casida 2006a); (Ford and Casida 2008)
	DIN-k (guanidine derivative fo DIN-h)	Mammals, plants	(Ford and Casida 2006a); (Ford and Casida 2008)
	DIN-l*, tetrahydrofuran carboxaldehyde, 3-Furfural	Mammals	(Ford and Casida 2006a)
	DIN-m, THFOL, tetrahydrofuran alcohol, 3-Furfuryl alcohol	Plants	(Ford and Casida 2008)
	DIN-n, THFCA, tetrahydrofuran-3-carboxylic acid	Mammals, plants	(Ford and Casida 2006a); (Ford and Casida 2008)
	DIN-p, 4-hydroxy-tetrahydrofuran-3-carboxylic acid	Mammals, plants	(Ford and Casida 2006a); (Ford and Casida 2008)
	DIN-r, THFMA, tetrahydrofuran-3-yl-methylamine	Mammals, plants	(Ford and Casida 2006a); (Ford and Casida 2008)
	446-DO, 1-[4-hydroxy-2-(hydroxymethyl) butyl]-3-methyl-2-nitroguanidine	Mammals, plants	FAO dinotefuran
	DIN-3-OH	Mammals, plants, water (photolysis)	FAO dinotefuran; USEPA 2004b
Imidacloprid	**IMI-olefin, olefin derivative, 4,5-dehydro-imidacloprid**	Honeybee, housefly, drosophila, mice	(Decourtye and Devillers 2010); (Suchail et al. 2001); (Nishiwaki et al. 2004); (Sparks et al. 2012); (Tomizawa and Casida 2003)
	**IMI-5-OH, 5-OH-imidacloprid, 5-hydroxy-imidacloprid, [(6-Chloro-3-pyridinyl) methyl]-4,5-dihydro-2-(nitroamino)-1H-imidazol-5-ol**	Honeybee, mice	(Decourtye and Devillers 2010); (Suchail et al. 2001); (Tomizawa and Casida 2003)
	**IMI-de**	Mice	(Tomizawa and Casida 2003)
	IMI-diol, 4,5-dihydroxy-imidacloprid	Honeybee, mice	(Suchail et al. 2001); (Tomizawa and Casida 2003)
	**IMI-NH, desnitro-imidacloprid**	Honeybee, plants, mice	(Suchail et al. 2001); (Tomizawa and Casida 2003)
	IMI-urea, urea derivative, N-((6-Chloropyridin-3-yl)-methyl)-imidazolidinone	Honeybee, mice	(Suchail et al. 2001); (Tomizawa and Casida 2003)
Imidacloprid, Nitenpyram, Acetamiprid, Thiacloprid	6-CNA, 6-chloronicotinic acid	Animals, plants, soil	(Suchail et al. 2001); (Nishiwaki et al. 2004); (Sparks et al. 2012); (Ford and Casida 2008); (Casida 2011); (Brunet et al. 2005); FAO acetamiprid; Lazic 2012; (Tokieda et al. 1999); (Phugare and Jadhav 2013); (Ford and Casida 2006b); FAO thiacloprid
Nitenpyram	NIT-COOH	Mice	(Ford and Casida 2008); (Casida 2011)
	NIT-deschloropyridine	Mice	(Ford and Casida 2008); (Casida 2011)
	NIT-dm, N-desmethyl nitempyram	Mice	(Ford and Casida 2008); (Casida 2011)
	NIT-CN	Mice	(Ford and Casida 2008); (Casida 2011)
	NIT-deschloropyridine derivatives	Mice	(Ford and Casida 2008); (Casida 2011)
Acetamiprid	Acetamiprid-D-desmethyl, N-desmethyl acetamiprid, IM-2-1, ACE-dm, N-(6-Chloro-3-pyridylmethyl)-N′-cyano-acetamidine	Animal, plants, soil (microbial)	FAO acetamiprid; (Brunet et al. 2005); (Casida 2011); (Ford and Casida 2008); (Chen et al. 2008); (Wang et al. 2012); (Wang et al. 2013a)
	IM-1-3, N-[(6-chloro-3-pyridyl)methyl]-N-methylacetamide, ACE-acet, ACE-urea	Animal, plants, soil, water (hydrolysis)	(Casida 2011); FAO acetamiprid; (Brunet et al. 2005); (Dai et al. 2010); (Liu et al. 2011)
	IM-2-3, N-[(6-chloro-3-pyridyl)methyl]acetamide, ACE-dm-acet	Mice, plants	(Casida 2011); FAO acetamiprid
	IM-1-2, N2-carbamoyl-N1- [(6-chloro-3-pyridyl)methyl]-N1-methylacetamidine, ACE-NCONH2	Mice, plants, soil (microbial)	(Casida 2011); FAO acetamiprid; (Phugare and Jadhav 2013)
	IM-2-2, N2-carbamoyl-N1- [(6-chloro-3-pyridyl)methyl]-acetamidine, ACE-dm-NCONH2	Mice, plants	(Casida 2011); (Ford and Casida 2008)
	IM-1-4, N-methyl(6-chloro-3-pyridyl)methylamine, N-methylpyridinylmethylamine	Animal (honeybees), plants, soil	(Casida 2011); (Ford and Casida 2006b); (Brunet et al. 2005); FAO acetamiprid; (Dai et al. 2010); (Liu et al. 2011); (Wang et al. 2013b); Tokieda 1999; (Phugare and Jadhav 2013); (Wang et al. 2013a)
	IM-0, (6-chloro-3-pyridyl)methanol, CPOL	Animal (honeybees), plants	(Brunet et al. 2005); FAO acetamiprid
	ACE-NH, descyano derivative	Plants, soil	(Casida 2011); (Wang et al. 2013a)
	IM-2-5, N1-(6-Chloropyridin-3-ylmethyl)-acetamidine, ACE-dm-NH	Animals	FAO acetamiprid
	IM-2-4, (6-chloro-3-pyridyl)methylamine, chloropyridinylmethylamine	Mice, plants	(Casida 2011); (Ford and Casida 2006a); (Ford and Casida 2008)
	N-methylpyridinylmethylamine	Soil	(Phugare and Jadhav 2013)
	(E)-1-ethylideneurea	Soil	(Phugare and Jadhav 2013)
	ACE-w, N′-cyano-N-methylacetimidamide	Mice, plants	(Casida 2011); (Ford and Casida 2006b); (Ford and Casida 2008)
	ACE-u, N′-cyanoacetimidamide	Mice, plants	(Casida 2011); (Ford and Casida 2006b); (Ford and Casida 2008)
Thiacloprid	THI-NH, M29, thiacloprid thiazolidinimine, 3-[(6-Chloro-3-pyridinyl)methyl]-2-thiazolidinimine, descyano derivative	Mice, plants, soil	(Ford and Casida 2006b); FAO thiacloprid; (Ford and Casida 2008)
	THI-ole, M38, thiacloprid-olefin, {3-[(6-chloro-3-pyridinyl)methyl]-2-thiazolylidene}cyanamide	Mice, plants	(Ford and Casida 2006b); FAO thiacloprid; (Ford and Casida 2008)
	THI-ole-NH	Mice, plants	(Ford and Casida 2006b); (Ford and Casida 2008)
	THI-4-OH, 4-hydroxy-thiacloprid, {3-[(6-chloro-3-pyridinyl)methyl]-4-hydroxy-2-thiazolidinylidene}cyanamide	Animals, plants, soil (microbial)	(Ford and Casida 2006b); (Ford and Casida 2008); FAO thiacloprid; (Zhao et al. 2009)
	Thiacloprid-amide, THI-NCONH2, 3-[(6-chloro-3-pyridinyl)methyl]-2-thiazolidinylidene}urea, M02	Mice, plants, Soil (microbial)	(Ford and Casida 2006b); (Ford and Casida 2008); FAO thiacloprid; (Dai et al. 2010)
	THI-4-OH-NCONH2, M37, {3-[(6-chloro-3-pyridinyl)methyl]-4-hydroxy-2-thiazolidinylidene}urea	Mice, plants	(Ford and Casida 2006b); (Casida 2011); (Ford and Casida 2008); FAO thiacloprid
	THI-SO	Mice, plants	(Ford and Casida 2006b); (Ford and Casida 2008)
	THI-SO3-H-NCONH2, Thiacloprid sulfonic acid, M30	Mice, plants, Soil	(Ford and Casida 2006b); (Ford and Casida 2008); PPDB 2013; FAO thiacloprid
	THI-SOMe	Mice	(Ford and Casida 2006b)
Cycloxaprid	CYC-OH, hydroxy derivatives	Mice	(Shao et al. 2013b)
	CYC-(OH)_2_, dihydroxy derivatives	Mice	(Shao et al. 2013b)
	CYC-NO, nitroso derivative	Mice	(Shao et al. 2013b)
	CYC-NH_2_, amine derivative	Mice	(Shao et al. 2013b)
	NMI, nitromethylene imidazole	Mice	(Shao et al. 2013b)
	NMI-NO, nitroso derivative of NMI	Mice	(Shao et al. 2013b)

*not observed

**Table 3:** First-generation metabolites of fipronil in various media and organisms. Metabolites known to be active toward invertebrates or mammals are highlighted in bold

Parent compound	Metabolites	Formation medium	Reference
Fipronil	Fipronil detrifluoromethylsulphinyl, 5-amino-3-cyano-1-(2,6-dichloro-4-trifluoromethylphenyl) pyrazole, MB 45897	Mammals, soil, plants (photolysis)	FAO fipronil, (Hainzl and Casida 1996); (France 2005)
	**Fipronil-sulfide, 5-amino-1-.[2,6-dichloro-4-(trifluoromethyl)phenyl]-4-[(trifluoromethyl)thio]-1H-pyrazole-3-carbonitrile, MB45950**	Mammals, soil, plants, water (photolysis)	FAO fipronil; (Bobé et al. 1998); (Aajoud et al. 2003); (France 2005); (Gunasekara et al. 2007)
	**Fipronil-sulfone, 5-amino-1-[2,6-dichloro-4-(trifluoromethyl)phenyl]-4-[(trifluoromethyl)sulfonyl]-1H-pyrazole-3-carbonitrile,** **MB 46136**	Mammals (milk), hens (eggs), soil, plants, water (incl. photolysis)	(Hainzl and Casida 1996); (Hainzl et al. 1998); (Bobé et al. 1998); FAO fipronil, (Tingle et al. 2003); (Aajoud et al. 2003); (France 2005)
	**Fipronil-desulfinyl, desthiofipronil, 5-amino-1-[2,6-dichloro-4-(trifluoromethyl)phenyl]-4-[(1R,S)-(trifluoromethyl)]-1H-pyrazole-3-carbonitrile, MB 46513**	Soil, plants, water (photolysis)	(Hainzl and Casida 1996); (Hainzl et al. 1998); (Bobé et al. 1998); FAO fipronil; (Tingle et al. 2003); (Aajoud et al. 2003); (Gunasekara et al. 2007)
	5-amino-3-cyano-1-(2,6-dichloro-4-trifluoromethylphenyl)-pyrazole-4-sulfonic acid, RPA104615	Soil, water (photolysis)	(Tingle et al. 2003); FAO fipronil
	5-amino-3-carbamyl-1-(2,6-dichloro-4-trifluoromethylphenyl)-4-trifluoromethylsulfonylpyrazole, RPA105320	Soil, plants	FAO fipronil
	**Fipronil-amide, 5-amino-3-carbamyl-1-(2,6-dichloro-4-trifluoromethylphenyl)-4-trifluoromethylsulfinylpyrazole, RPA 200766**	Mammals, soil, plants, water (hydrolysis)	(Bobé et al. 1998); (Tingle et al. 2003); (Aajoud et al. 2003); FAO fipronil
	5-amino-3-carbamyl-1-(2,6-dichloro-4-trifluoromethylphenyl)-4-trifluoromethylsulfinylpyrazole-3-carboxylic acid, RPA 200761	Mammals, soil, plants, water	FAO fipronil; (France 2005)
	Various conjugates in urine and bile (RPA 105048, UMET/10, UMET/3, FMET/9, UMET/4, FMET/7, FMET/10, UMET/15)	Mammals	FAO fipronil; (France 2005)
	MB 46400	Mammals, hens (eggs)	FAO fipronil; (France 2005)
	RPA 108058	Mammals, hens (eggs)	FAO fipronil
	Ring-opened 106889	Mammals, hens (eggs)	FAO fipronil
	RPA 106681	Soil	FAO fipronil

### Thiamethoxam, clothianidin, and dinotefuran

#### Animals

The metabolism of thiamethoxam (hereafter also TMX) is closely related to that of clothianidin (hereafter also CLO). As a result, thiamethoxam produces both metabolites in common with clothianidin as well as some specific metabolites (Ford and Casida 2006a). The main metabolic pathways of thiamethoxam involve hydroxylation at the oxadiazine part of the molecule, accompanied by ring opening, leading to the production of clothianidin, its principal intermediate in mammals, insects, and plants (Nauen etl al. 2003; Ford and Casida 2006a; Karmakar et al. 2009; Casida 2011). Other metabolic pathways of both TMX and CLO are N-demethylation and/or nitro reduction reactions (Ford and Casida 2006a; Casida 2011; Kim et al. 2012), leading to TMX-dm and CLO-dm or their N-nitroso- or N-amino-guanidines derivatives. These are two metabolites with toxicity comparable to those of the parent compounds, maintaining almost unaltered binding affinity to the nAChR (Chen et al. 2005; Ford and Casida 2006a). In fact, N-desmethyl thiamethoxam is almost as active as the insecticide imidacloprid (Karmakar et al. 2009). However, nitro reduction reverses the relative toxicity to insects and mammals, being a mechanism of detoxification for insects and bioactivation for mammals (Kanne et al. 2005; Honda et al. 2006, Casida 2011).

Thiamethoxam has been found to be a liver carcinogen in mice (*M. musculus*) (Green et al. 2005a, 2005b; Tomizawa and Casida 2005). Green et al. (2005a, b) proposed that TMX-dm may be a hepatotoxicant. This suggests that contrary to initial ideas, neonicotinoids may significantly affect the health of vertebrates including humans. A detailed review of such effects is, however, outside the scope of the present review.

Further steps in the metabolism pathway involve either phase I metabolites (N-methylene and C-methylene hydroxylation) leading to a wide range of nitroguanidine (NG) and chlorothiazolylmethyl (CTM) cleavage products or oxidation to the urea derivatives (TMX-Urea, TMX-dm. Urea, CLO-Urea, CLO-dm-Urea) or phase II metabolites by adding pyruvate to give the methyltriazinones (TMX-dm-tri, CLO-tri, and CLO-dm-tri) (Chen et al. 2005; Ford and Casida 2006a).

While all CTM cleavage products are in common between thiamethoxam and clothianidin, only some NG cleavage products are in common between the two insecticides (methylnitroguanidine (NG-E), methylguanidine (NG-F), and other NG compounds) (Yokota et al. 2003; Ford and Casida 2006a; Kim et al. 2012). Other NG metabolites are specific to thiamethoxam (NG-A, NG-B, NG-C, and NG-D). These compounds may continue their metabolism leading to a wide range of breakdown products.

Most of the metabolites of thiamethoxam and clothianidin are observed not only in small mammals, such as mice and rats, but also in dogs and hens (USEPA 2000; Klein 2003; USEPA 2003b; Yokota et al. 2003; USEPA 2004a; Ford and Casida 2006a; Kim et al. 2012).

Dinotefuran differs from TMX and CLO by its tetrahydrofuranyl moiety instead of the chlorothiazolyl part. As for thiamethoxam and clothianidin, the principal metabolic pathways of dinotefuran (hereafter also DIN) in mammals involve N-demethylation, nitro reduction, and N-methylene hydroxylation accompanied by amine cleavage (Ford and Casida 2006a; Casida 2011). Common metabolites have been described (NG-E, NG-F, and other NG compounds) (FAO dinotefuran). The metabolism of dinotefuran differs from that of clothianidin and thiamethoxam by the ready hydroxylation and metabolism of the tetrahydrofuranyl moiety. The pharmacokinetics of dinotefuran are characterized by a rapid metabolism and excretion probably associated with its high polarity and fast metabolism of the hydrofuranyl moiety (Ford and Casida 2006a). As a result, DIN metabolites follow a similar pattern than those of TMX and CLO (DIN-dm, DIN-NNO, DIN-dm-NNO, DIN-NNH2, DIN-dm-NNH2, DIN-NH, DIN-dm-NH) and urea derivatives. Phase II metabolism, with pyruvate addition, produces methyltriazinones (DIN-tri and DIN-dm-tri) (Ford and Casida 2006a; Casida 2011). As already observed for thiamethoxam and clothianidin, the nitro reduction pathway causes a shift from insect-selective to vertebrate-selective action (Kanne et al. 2005; Honda et al. 2006; Casida 2011).

The tetrahydrofuran group may undergo metabolization including hydroxylation at 2, 5, and 4 positions, ring opening, N-acetylation, N-demethylation or nitro reduction (Ford and Casida 2006a).

Most of the metabolites are observed in both small mammals such as mice and rats but also in dogs and hens (Ford and Casida 2006a; USEPA 2003c; USEPA 2004b). Hydrolysis of the tetrahydrofuran ring to form 1-[4-hydroxy-2-(hydroxymethyl) butyl]-3-methyl-2-nitroguanidine (446-DO) has also been reported (FAO dinotefuran).

#### Plants

Clothianidin metabolism in plants has been evaluated in a variety of crops, including maize, sugar beet, fodder beet, apples, and tomatoes (EFSA 2010). Metabolism of thiamethoxam has been evaluated in maize, rice, pears, cucumbers, lettuce, and potatoes (FAO thiamethoxam). The plant enzymes responsible for the conversion of thiamethoxam and clothianidin into their metabolites have not been examined so far (Ford and Casida 2008).

Phase I metabolites in spinach, maize, and sugar beet were remarkably similar to those observed in small mammals (Chen et al. 2005; Ford and Casida 2006a, 2008), with the main metabolic pathways proceeding through N-demethylation and nitro reduction (FAO thiamethoxam; Ford and Casida 2008).

Thiamethoxam is rapidly metabolized to clothianidin in cotton plants, while TMX-dm is not significantly produced (Karmakar et al. 2009). EFSA (2010) describes clothianidin as being metabolized extensively in the leaves predominantly leading to CLO-NH and NG-F (Kim et al. 2012). Clothianidin is oxidatively cleaved in plants to the carboxylic acid derivative, among other metabolites and cleavage products (Ford and Casida 2008; Ford et al. 2010; FAO clothianidin). In spinach, thiamethoxam, clothianidin, and their N-demethylated products form nitrosoguanidine, guanidine, and urea derivatives (Ford and Casida 2008; FAO thiamethoxam; FAO clothianidin). Conjugated products from thiamethoxam and clothianidin have not been observed in spinach and neither have methylthio derivatives (Ford and Casida 2008). Contrary to the metabolism in mammals, clothianidin undergoes hydroxylation at the inner guanidine nitrogen atom leading to the N-OH derivative (N-2-chlorothiazol-5-ylmethyl-N-hydroxy-N′-methyl-N″-nitroguanidine, THMN) followed by glycosylation (phase II metabolism) in maize, apple, and sugarbeet (FAO clothianidin).

Metabolism of dinotefuran in plants is similar to that in mammals, leading mainly to methylguanidine, nitroguanidine, and urea metabolites (Ford and Casida 2008; Casida 2011; Rahman et al. 2013; FAO dinotefuran). As for clothianidin, N-methylene hydroxylation yields either tetrahydrofurylmethylamine (THFMA/DIN-r), which could be further metabolized through phase I (e.g., N-acetylation, oxidation, reduction…) and/or phase II (glucoside derivative) reactions (Ford and Casida 2008). In plants, internal ring formation yields 6-hydroxy-5-(2-hydroxyethyl)-1-methyl-1,3-diazinane-2-ylidene-*N*-nitroamine (PHP). NG-E and NG-F are observed as major cleavage products (Ford and Casida 2008; FAO dinotefuran).

#### Water

In water, thiamethoxam is stable to hydrolysis in dark conditions at pH 1–7 (De Uderzo et al. 2007) while it is quickly hydrolyzed at pH 9 and 20 °C (European Commission 2006) and almost completely degraded (ca. 96 %) under UV radiation in about 10 min (De Uderzo et al. 2007). The main hydrolysis products are identified: TMX-Urea, clothianidin and its derivatives (N-(2-chlorothiazol-5-ylmethyl)-N′-nitrourea (CTNU), CTM-i, methylurea (MU), and NG-B) (FAO thiamethoxam).

Conversely, De Uderzo et al. (2007) proposed a photodegradation mechanism of thiamethoxam to form the guanidine derivatives (TMX-NH), with a loss of HNO3. After that, a nucleophilic substitution of the Cl with OH in the thiazolic ring could occur, which then quickly decomposes to 5-methyl-2(3H)-thiazolone and NG-F (De Uderzo et al. 2007). 5-Methyl-2(3H)-thiazolone could in turn decompose to volatile products, such as carbonyl sulfide and isocyanic acid, already observed by Schwartz et al. (2000). Other observed photodegradation products include an oxazine derivative, possibly formed by extrusion of S to generate an azetidinone intermediate, and an acrylonitrile derivative from hydrolysis of the imine group of the oxazol ring (De Uderzo et al. 2007).

No peer-reviewed literature could be found concerning clothianidin breakdown in water. However, the FAO mentions that this compound degrades by hydrolysis and/or photolysis into CLO-Urea, with further cleavage to methylurea (MU) and 2-chlorothiazol-5-yl-methylamine (ACT), (FAO clothianidin). Clothianidin could also be hydrolyzed to the nitro urea derivative (CTNU) and further cleaved into ACT. Nitro reduction, cleavage at the methylene bridge or complex cyclization reaction accompanied by loss of nitro group, chlorine elimination, and desulphuration convert the parent compound into CLO-NH, NG-F and forms 7-methylamino-4H-imidazo[5,1-b][1,2,5]thiadiazin-4-one (MIT). Successively, ring cleavage forms 2-methylamino-2-imidazolin-5-one (MIO), 4-hydroxy-2-methylamino-2-imidazolin-5-one (HMIO), NG-F and formamide (FA) with a final mineralization to carbon dioxide (FAO clothianidin).

Hydrolysis of dinotefuran in dark conditions and alkaline pH produces DIN-Urea. Photolysis on surface water produces DIN-Urea, DIN-NH, DIN-2-OH, and DIN-3-OH (USEPA 2004b).

#### Soil

No peer-reviewed literature could be found concerning thiamethoxam breakdown in soil. However, the FAO provides some information on this regard (FAO thiamethoxam). The metabolic pathways of thiamethoxam in soil, under aerobic conditions, lead to the conversion of TMX into CLO, which then is degraded to CLO-NH and CLO-Urea. CLO-dm is also observed as a degradation product. Nitro reduction of the parent compound also occurs, which finally forms TMX-Urea. The intermediate TMX-NH has been observed only in rice-paddies so far. NG-A cleavage product, from N-methylene hydroxylation, has also been observed as a major product in soil (FAO thiamethoxam). The main metabolite formed in anaerobic conditions is TMX-NH but TMX-Urea has been also observed (European Commission 2006).

The aerobic degradation of clothianidin in soil proceeds through three main pathways. The first pathway starts with N-demethylation of clothianidin to form CLO-dm and N-methylene hydroxylation to form nitroguanidine (NG-G). The second pathway starts with the N-methylene hydroxylation to form NG-F and proceeds through N-demethylation to form NG-G. A third route involves the formation of CLO-Urea via nitro reduction (FAO clothianidin). The metabolisation of clothianidin further progresses to carbon dioxide.

In soil incubated under aerobic conditions in the dark at 20 °C, dinotefuran degraded to NG-E and NG-F as major degradation products. Other minor observed metabolites were DIN-Urea and DIN-dm (FAO dinotefuran). Dinotefuran and its metabolites are further mineralized to carbon dioxide. It has been also found that photolysis is not a significant degradation pathway of dinotefuran in soil (FAO dinotefuran). DIN-NH has been observed in soil under anaerobic conditions (USEPA 2004b).

### Imidacloprid and nitenpyram

#### Animals (and plants)

The metabolic pathways of neonicotinoids present many similarities between insects and plants. In the honey bee, imidacloprid (hereafter also IMI) is transformed mainly to olefin, 5-hydroxy-imidaclorpid (5-OH-imidacloprid), 4,5-dihydroxy-imidacloprid, desnitro-imidacloprid, urea derivative, and 6-chloronicotinic acid (6-CNA). Among these metabolites, olefin and 5-OH-imidacloprid exhibit toxicity both in acute and chronic exposures (Suchail et al. 2001). Thus, the biotransformation of imidacloprid leads to a metabolic activation and to the concentration of the toxic metabolites in the brain and thorax of the honey bee for more than 96 h (Suchail et al. 2004a, 2004b). This results in a metabolic relay, in which imidacloprid induces first toxicity and then the toxic metabolites act in bees surviving the early action of imidacloprid. This leads to a lethal phenomenon that lasts more than 96 h, contrary to the other neurotoxic insecticides for which the maximum mortality rate is generally observed between 10 and 24 h (Suchail et al. 2001). The metabolism of imidacloprid is very similar in bees and flies with hydroxylated imidacloprid derivatives, olefin, 6-CNA, and the imidazoline moiety as main metabolites in the housefly and drosophila (Nishiwaki et al. 2004; Sparks et al. 2012). This suggests that insects may exhibit close neonicotinoid metabolic pathways. Thus, metabolic activation and sensitivity to certain plant metabolites might be a common feature in insects. That could be the reason for which the conserved toxicity profiles have been depicted in bees and in flies after chronic exposure to concentrations three to five orders of magnitude lower than LC_50_ (Charpentier et al. 2014).

Much of the use of neonicotinoids takes advantage of the systemic properties of the active substances and involves plant treatments by seed dressing. As a result, humans and animals are exposed through consumption of vegetables containing neonicotinoid active substances taken up by plants, and their metabolites. Exposure through food should be taken into account, since studies have shown that nicotine and nicotine derivatives, such as the neonicotinoids imidacloprid, acetamiprid, and clothianidin, can be rapidly and efficiently absorbed through the intestine barrier (Yokota et al. 2003; Brunet et al. 2004; Brunet et al. 2008). Moreover, seven metabolites of these neonicotinoids have been found in human urine of sick patients (Taira et al. 2013). Among plant metabolites, desnitro-imidacloprid is of particular interest because it displays high toxicity to vertebrates associated with an agonist action on the α4β2 nAChRs (Chao and Casida 1997; D'Amour and Casida 1999; Tomizawa and Casida 2000; Tomizawa et al. 2001a). Desnitro-imidacloprid is also able to activate intracellular calcium mobilization and the extracellular signal-regulated kinase cascade through its interaction with the nAChR (Tomizawa and Casida 2002). In mice, imidacloprid is biotransformed into IMI-de, IMI-olefin, IMI-NH (desnitro-imidacloprid), IMI-urea, IMI-urea-gluc, IMI-urea-gent, IMI-diol, IMI-diol-gluc, IMI-5-OH, IMI-5-OH-gluc, IMI-NNO, 6-CNA and different imidazoline and pyridinyl derivatives. IMI-NH is generated by the action of cytochromes P450 on imidacloprid (Tomizawa and Casida 2003). The appearance of this metabolite can be considered a bioactivation, since IMI-NH exhibits toxicity to mammals due to its ability to bind to α4β2 nAChR (Chao and Casida 1997; D'Amour and Casida 1999; Tomizawa and Casida 2000; Tomizawa et al. 2001a; Tomizawa and Casida 2003, 2005).

However, desnitro-imidacloprid is a detoxification derivative in insects. The 6-CNA is a metabolite common to chloropyridinyl neonicotinoids (Ford and Casida 2008; Casida 2011). Thus, the risk posed by 6-CNA to the honey bee might be common to the use of imidacloprid, thiacloprid, acetamiprid, and nitenpyram.

Nitenpyram (hereafter also NIT) is metabolized in mice into NIT-COOH, NIT-deschloropyridine, NIT-dm (N-desmethyl nitempyram), NIT-CN, and different NIT-deschloropyridine derivative (Ford and Casida 2008; Casida 2011). The NIT metabolites have not been subjected to in-depth toxicological investigations. These metabolites can undergo an oxidation of the cyano group into a carboxylic acid (Ford and Casida 2008; Casida 2011).

#### Soil and water

Further to metabolites described for plants and animals, desntiro-olefin, 2,5 diketone, carbone dioxide, and 6-hydroxynicotinic acid have been described in soil (FAO imidacloprid).

### Acetamiprid and thiacloprid

#### Animals

In mammals, acetamiprid (hereafter also ACE) undergoes a rapid and efficient intestinal absorption (Brunet et al. 2008). As for the other neonicotinoids, N-demethylation is the main metabolisation pathway for acetamiprid and thiacloprid (hereafter also THI). In insects, acetamiprid undergoes a rapid biotransformation, which signals a high metabolic activity, being metabolized into IM2-1 (ACE-dm), IM1-3 (ACE-urea), IM1-4 (*N*-methyl-chloropyridinylmethylamine), IM0 (6-chloropicolyl alcohol), IC0 (6-CNA) and two unknown metabolites (Brunet et al. 2005; Ford and Casida 2006a; Casida 2011). The metabolite 6-CNA remains stable for more than 72 h in all biological compartments, except gut-free abdomen, which could explain the toxicity of acetamiprid (Brunet et al. 2005). Thiacloprid is transformed into THI-NH, THI-ole, THI-ole-NH (putative), THI-4-OH, THI-NCONH_2_, THI-4-OH-NCONH_2_, THI-SO, THI-SO_3_H-NCONH_2_, and THI-SMe (Ford and Casida 2006b; Casida 2011). Descyano-thiacloprid (THI-NH) is generated by the action of cytochromes P450 on thiacloprid in vivo (Tomizawa and Casida 2003, 2005). As for imidacloprid and desnitro-imidacloprid, the appearance of THI-NH can be considered as thiacloprid bioactivation because THI-NH exhibits a toxicity to mammals in relation with its ability to bind to α4β2 nicotinic acetylcholine receptors (Chao and Casida 1997; D'Amour and Casida 1999; Tomizawa and Casida 2000; Tomizawa et al. 2001a; Tomizawa and Casida 2003, 2005). In insects, THI-NH is instead a detoxification metabolite.

#### Plants

As seen for the other neonicotinoids, metabolization of acetamiprid and thiacloprid is similar in plants and mammals. Acetamiprid metabolization involves several initial sites of attack: N-demethylation, cyano hydrolysis, cleavage of 6-CNA. Additionally, cleavage of N-CN linkage from acetamiprid, which yields the N-descyano compound (ACE-NH) also occurs (Ford and Casida 2008; Casida 2011).

Thiacloprid metabolization involves five different sites of attack: cyano hydrolysis (THI-NCONH2), sulfoxidation (THI-SO, THI-SO3H-NCONH2), hydroxylation at the 4-position (THI-4-OH, THI-4-OHNCONH2), conversion to the olefin (THI-ole) and loss of the cyano group (THI-NH, THI-ole-NH). The urea derivative (THI-4-OHNCONH2) and THI-SO were the major metabolites observed (Ford and Casida 2008; Casida 2011).

#### Soil and water

Acetamiprid is stable to hydrolysis and photolysis, the main metabolite in soil being IM1-4 (FAO acetamiprid; Dai et al. 2010; Liu et al. 2011; Wang et al. 2013a; Wang et al. 2013b). Minor metabolites are ACE-urea and 6-CNA (FAO acetamiprid; Dai et al. 2010; Liu et al. 2011). Biotransformation of acetamiprid produces the N-demethylated derivative (Chen et al. 2008; Wang et al. 2012). Recently, Phugare and Jadhav (2013) evidenced the formation of ACE-NCONH2 from microbial degradation in soil, which is then cleaved to N-methylpyridinylmethylamine and (E)-1-ethylideneurea with further oxidative cleavage to 6-CNA.

Thiacloprid is stable to hydrolysis (95–98 % recovery after 30 days). It can be degraded to THI-NCONH2 in soil in both light and dark conditions (FAO thiacloprid), which can be further transformed into THI-NH and THI-SO3-H-NCONH2.

### *Cis*-neonicotinoids and new-generation insecticides

Cycloxaprid, paichongding, imidaclothiz, and sulfoxaflor are newly developed neonicotinoid-like insecticides. Paichongding and cycloxaprid are *cis*-neonicotinoids (Li et al. 2011; Shao et al. 2011; Cui et al. 2012), imidaclothiz is a nitroguanidine thiazole neonicotinoid (Wu et al. 2010), and sulfoxaflor is a sulfoximine insecticide, whose insecticidal activity could be closely related to its very high efficacy at nAChRs (Watson et al. 2011). However, only a few studies have been published on the metabolism of these new substances in insects and mammals.

#### Animals

Cycloxaprid (hereafter also CYC) metabolism has been investigated in mice (Shao et al. 2013b). Five monohydroxy (CYC-OH) and one dihydroxy (CYC-(OH)_2_) metabolites have been characterized, along with compounds resulting from modification of the NO_2_ group into nitroso and amine derivatives (CYC-NO and CYC-NH_2_, respectively). The next more abundant product was nitromethylene imidazole (NMI) and its NO derivative (NMI-NO). When they bind to housefly (*M. domestica* L.) head membranes, NMI and CYC exhibit dissociation constants of 1.1 and 28 nM, respectively. This indicates that, as imidacloprid, the degradation of CYC generates toxic metabolites with high affinity for receptors. As a result, metabolites could prolong their toxic effects. Should these metabolites be found on plants, insect exposure could occur.

Sulfoxaflor metabolism has been investigated in vitro on drosophila D.mel-2 cells transfected with CYP6G1 (Sparks et al. 2012). Compared to imidacloprid, acetamiprid, dinotefuran, thiamethoxam, and clothianidin for which the extents of metabolism are respectively 85.1, 95.5, 55.1, 46.8, and 45.6 % after 24 h, sulfoxaflor presents an almost undetectable metabolism. These results could explain the absence of cross-resistance to sulfoxaflor in insects resistant to neonicotinoids or other insecticides. However, because sulfoxaflor metabolism has been investigated only with CYP6G1, the extrapolation of the least metabolic susceptibility to the whole drosophila metabolism is difficult.

### Fipronil

#### Animals

In mammals, fipronil can be metabolized at its trifluoromethylsulfinyl or cyano moieties through three major pathways: (1) oxidation at the sulfinyl moiety to form fipronil-sulfone; (2) reduction at the sulfinyl moiety yielding fipronil-sulfide; and (3) by hydrolysis of the cyano moiety to form fipronil-amide followed by further hydrolysis to the corresponding carboxylic acid (5-amino-1-(2,6-dichloro-4-trifluoromethylphenyl)-4-trifluoromethylsulfinyl pyrazole-3- carboxylic acid) (France 2005).

Metabolism in rats has proved to be independent of dose level, regime, and sex (France 2005). In the rat, two urinary metabolites have been identified following deconjugation with glucuronidase and sulfatase, leading to pyrazole ring-opened compounds. Other compounds can be found in urine as the derivates fipronil-amide, fipronil-sulfone, and fipronil-sulfide, and the metabolite of fipronil-sulfone, defluoromethylsulfynil-fipronil (France 2005; FAO fipronil). Fipronil itself can also be found in urine. Fipronil-sulfone is the major metabolite and often the only one found in the tissues of the species examined: fat, adrenal gland, pancreas, skin, liver, kidney, muscle, thyroid, and ovaries and uterus, as well as in foodstuffs: milk and eggs (FAO fipronil). Fipronil, and its amide, sulfone, and sulfide derivates are the main compounds recovered from fat tissues, consistently with their lipophilic nature. Fipronil and its amide, sulfone, and sulfide derivates are the main components found in feces, together with seven other metabolites found at minimal quantities. At least 16 different derivates are present in bile, including the fipronil-carboxylic acid metabolite (FAO fipronil).

Experiments on rats, goats, and hens with the photolytic metabolite of fipronil, desulfinyl-fipronil, yield numerous urinary metabolites mainly as a result of phase II metabolism. These metabolites result from the metabolism of radicals of the pyrazole ring different from the trifluoromethylsulfinyl or cyano moieties. Among others, the following have been described: (1) N-sulfate conjugate of desulfinyl-fipronil, (2) two amino acid conjugates resulting from the action of deconjugating enzymes glucuronidase and sulfatase followed by acidic hydrolysis, (3) 5-aminoglucuronide confugate, (4) 5-(*N-*cysteinyl) conjugate of fipronil-desulfinyl, and (5) a 4-cyano- 5-(*N*-cysteinylglycine) conjugate, (4) and (5) linked through the cysteine residue. Metabolization of desulfinyl-fipronil leads to the amide derivate, 4-cyano-5-(–cysteinyl) derivate, which in turn may result in the 4-carboxylic acid-fipronil (Totis 1996 in FAO fipronil). Ring-opened conjugates were observed in goat’s liver (Johnson et al. 1996 in FAO fipronil).

#### Plants

Translocation studies carried out with [^14^C]fipronil on maize, sunflower, and sugar beet show uptake of about 5 %. Fipronil could be co-formulated with numerous polymers in order to enhance the systemicity of this active substance (Dieckmann et al. 2010c). Studies carried out in potatoes, rice, sunflower, sugar beet, cabbage, cotton, maize, showed metabolism of the mother compound in plants via hydrolysis to amide-fipronil, oxidation to the sulfone-fipronil and reduction to the sulfide-fipronil. Foliar application was also subject of photodegradation to desulfinyl-fipronil. Fipronil-sulfone can undergo photolysis resulting in sulfonic acid (Roberts and Hutson 1999). This molecule may be target of cleavage and loss of the sulfone moiety, resulting in detrifluoromethylsulfinyl-fipronil. A carboxylic derivate of fipronil can be produced from the hydrolysis of the radical CONH_2_ of fipronil-amida (FAO fipronil).

Residues of fipronil, fipronil-amida, fipronil-sulfone, and fipronil-carboxylic acid, as well as minor undetermined derivates, have been found in boll components following seed dressing in cotton (France 2005). Fipronil and its desulfinyl and sulfone derivates have been found in pollen loads and honey (Bonmatin et al. 2007; Chauzat et al. 2011).

#### Soil and water

Fipronil degrades in water and soil through various metabolic pathways: (1) hydrolysis to the amide metabolite; (2) oxidation to fipronil-sulfone; and (3) reduction to fipronil-sulfide, mainly under anaerobic conditions (Raveton et al. 2007). Photolysis may also occur, leading to desulfinyl-fipronil and other aniline derivates (Raveton et al. 2006). A minor photoproduct both in water and soil surfaces is sulfonic acid. In aqueous surfaces, fipronil has proved to be stable in dark conditions. However, pH is a relevant factor determining metabolism. Hydrolysis kinetics at different pH values differ from half-lives of 770 h at pH 9 to 2.4 h at pH 12. Fipronil remains stable under acid (pH 5.5) and neutral conditions (Bobé et al. 1998). An amide derivate of the fipronil-sulfone can be present following hydrolysis or the cyano moiety (FAO fipronil), which can be further hydrolyzed rendering a carboxylic acid derivate. Photolysis of fipronil-sulfone results in the production of sulfonic acid. Fipronil-sulfide can follow hydrolyzes of its cyano moiety leading to a carboxylic acid derivate.

Detrifluoromethylsulfinyl-fipronil can appear in soil following cleavage of the trifluoromethylsulfinyl moiety (FAO fipronil).

Adsorption and leaching studies carried out in laboratory show that fipronil and its main metabolites are slightly mobile in soil (IUPAC 2014).

## Conclusion

This paper summarizes some of the key reasons for the success of neonicotinoids and fipronil and documents their rapidly expanding share of the global insecticide market in the last 25 years. Their physicochemical characteristics (extensively covered in Bonmatin et al. (2014)), especially in terms of water solubility, pKa, and *K*_ow_, confer systemic properties enabling them to be absorbed and translocated within all plant tissues. They are persistent (e.g., imidacloprid half-life in soil is ca. 6 months) and neurotoxic. Neonicotinoids share greater affinity toward arthropod nACh receptors than toward those of mammals and other vertebrates. Fipronil acts on insect specific receptors. This makes them highly efficient insecticides with reduced operator and consumer risk compared to some of their predecessors such as organophosphorous and carbamate insecticides. Furthermore, their mode of action enables new strategies for pest control that profit from the existing synergies between these substances and either other chemicals or microorganisms. As a result, there are a wide range of uses available, including seed coating and root bathing, as invertebrate pest control in agriculture, horticulture, orchards, forestry, veterinary applications, and fish farming. However, these same properties have led to problems. Specifically, their widespread (Main et al. 2014) and prophylactic use, their systemic properties in plants, their broad spectrum of toxicity in invertebrates, and the persistence and environmental fate of parent compounds and metabolites renders them potentially harmful to a broad range of non-target organisms. Subsequent papers in this review of the global literature explore different aspects of these risks. Pisa et al. (2014) and (Gibbons et al. (2014) extensively cover the potential impacts on non-target invertebrates and vertebrates, respectively. Chagnon et al. (2014) explore the risks of their large scale of use to ecosystem functioning and services. These papers show a growing body of evidence that persistent, low concentrations of these insecticides pose serious risks of undesirable environmental impacts (Tennekes and Sánchez-Bayo 2011; Roessink et al. 2013), and therefore the sustainability of the current heavy reliance upon these compounds is questionable considering the availability of existing alternative agricultural and forestry practices (Furlan and Kreutzweiser 2014).

## Notes

Authors are in the alphabetic order with the exception of the corresponding author. They declare no competing conflict of interest. All authors are working for public agencies or academic institutions, except NSD working both for the Utrecht University and the technical center CARI (mainly supported by public funds), VAR is employed by Buglife (a UK charity devoted to the conservation of invertebrates), DWG is employed by RSPB (a UK nature conservation charity), and DN is financed by the Stirling Triodos Fundation.
